# Guideline for the Management of Diabetes Mellitus in the Elderly in China (2024 Edition)

**DOI:** 10.1002/agm2.12294

**Published:** 2024-03-29

**Authors:** Lixin Guo, Xinhua Xiao

**Affiliations:** ^1^ National Center of Gerontology, Chinese Society of Geriatrics, Diabetes Professional Committee of Chinese Aging Well Association Beijing China; ^2^ Department of Endocrinology, Beijing Hospital, National Center of Gerontology, Institute of Geriatric Medicine Chinese Academy of Medical Sciences Beijing China; ^3^ Department of Endocrinology Peking Union Medical College Hospital, Chinese Academy of Medical Sciences Beijing China

**Keywords:** diabetes, guidelines, the older adults

## Abstract

With the deepening of aging in China, the prevalence of diabetes in older people has increased noticeably, and standardized diabetes management is critical for improving clinical outcomes of diabetes in older people. In 2021, the National Center of Gerontology, Chinese Society of Geriatrics, and Diabetes Professional Committee of Chinese Aging Well Association organized experts to write the first guideline for diabetes diagnosis and treatment in older people in China, the *Guideline for the Management of Diabetes Mellitus in the Elderly in China (2021 Edition)*. The guideline emphasizes that older patients with diabetes are a highly heterogeneous group requiring comprehensive assessment and stratified and individualized management strategies. The guideline proposes simple treatments and de‐intensified treatment strategies for older patients with diabetes. This edition of the guideline provides clinicians with practical and operable clinical guidance, thus greatly contributing to the comprehensive and full‐cycle standardized management of older patients with diabetes in China and promoting the extensive development of clinical and basic research on diabetes in older people and related fields. In the past 3 years, evidence‐based medicine for older patients with diabetes and related fields has further advanced, and new treatment concepts, drugs, and technologies have been developed. The guideline editorial committee promptly updated the first edition of the guideline and compiled the *Guideline for the Management of Diabetes Mellitus in the Elderly in China (2024 Edition)*. More precise management paths for older patients with diabetes are proposed, for achieving continued standardization of the management of older Chinese patients with diabetes and improving their clinical outcomes.

## Graphical abstract

Overall health assessment should be performed to formulate individualized glycemic goals and blood glucose control plans. If individualized glycemic goals fail to be achieved, hypoglycemic drugs should be added, besides lifestyle interventions. Therapeutic drug choices should be based on the health status of elderly patients and whether established/high risk factors of atherosclerotic cardiovascular disease (ASCVD), HF, or CKD are present.

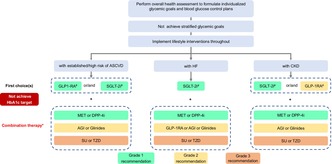

## 1 INTRODUCTION

China's society is aging. According to the data in the *Statistical Communiqué on the 2021 Civil Affairs Development* published by the Ministry of Civil Affairs of the People's Republic of China in August 2022, China had 267 million people 60 years or older, accounting for 18.9% of the total population, of whom 201 million were 65 years or older, accounting for 14.2% of the total population.^1^ According to international practice, people 65 years or older are defined as older adults, and patients 65 years or older with diabetes are defined as older patients with diabetes,^2^ including those diagnosed with diabetes before or after the age of 65 years. With the trends of aging in China and growing demands for diabetes management in older people, an urgent need exists to standardize the management of older patients with diabetes.

China's Medium‐to‐Long Term Plan for the Prevention and Treatment of Chronic Diseases (2017–2025), issued by the General Office of the State Council, highlights that, by 2025, the number of managed patients with diabetes will reach 40 million, and the standardized management rate of patients with diabetes is expected to reach 70%.^3^ The general principles of diabetes diagnosis and treatment may apply to older patients; however, older patients with diabetes require particular considerations regarding blood glucose management methods, goal setting, and principles of drug choice, given the high frequency of multiple complications and/or comorbidities, atypical symptoms, and poor self‐management, and the high risk of hypoglycemia. Therefore, an urgent need exists for standardizing and refining guidance for the management of the diagnosis and treatment of older patients with diabetes, to improve their clinical prognosis. Three authorities, the International Diabetes Federation, the American Diabetes Association, and the Endocrine Society, have published guidelines or consensus statements on diabetes in older people, which provide guidances and serve as references for the clinical diagnosis and treatment of diabetes in older people. In 2021, experts in diabetes, geriatrics, and related fields, organized by the National Center of Gerontology, the Chinese Society of Geriatrics, and the Diabetes Professional Committee of Chinese Aging Well Association, jointly compiled the *Guideline for the Management of Diabetes Mellitus in the Elderly in China (2021 Edition)*.^4^ In 2022, targeting type 2 diabetes mellitus (T2DM) in older people in China, researchers released the *Clinical Guidelines for Prevention and Treatment of Type 2 Diabetes Mellitus in the Elderly in China (2022 Edition)*.^5^ Those guidelines have played important roles in promoting the standardized diagnosis and treatment of diabetes in older people in China.

In the past 3 years, extensive research results have emerged in the field of diabetes mellitus in older people; new medications for controlling diabetes mellitus and its complications have been introduced; and evidence from clinical studies has accumulated. Consequently, the guideline editorial committee updated the *Guideline for the Management of Diabetes Mellitus in the Elderly in China (2021 Edition)* and released the *Guideline for the Management of Diabetes Mellitus in the Elderly in China (2024 Edition)*.

The guideline, like the previous version, discusses issues associated with management of diabetes mellitus in older people in the greatest detail possible, focusing on aspects that significantly affect patients' overall health status and quality of life, and differs from the general considerations for adults with diabetes mellitus; these aspects include epidemiology of diabetes and its complications in older people; clinical characteristics of diabetes in older people; comprehensive geriatric assessment (CGA); and management of blood glucose, atherosclerotic cardiovascular disease (ASCVD) risk factors, and acute and chronic complications. The management of risk factors for ASCVD, acute and chronic complications, as well as the management of comorbidities and special conditions in older patients with diabetes are discussed in depth. The guideline also highlights the need for stratified and highly individualized management strategies, given the considerable heterogeneity among older patients with diabetes. In addition, the guideline continues to recommend the simple treatment concept and the de‐intensified treatment strategy proposed in the prior version. In this update, the editorial committee searched for and organized evidence regarding diabetes in older people over the past 3 years, paying particular attention to evidence regarding diabetes in older people in China. This version of the guideline provides specific scales for comprehensive health assessment, which are more clinically practical, and recommends anti‐hyperglycemic drug treatment pathways for older patients with diabetes with various complications or comorbidities and related risks, thus further advancing the concept of clinical outcome‐oriented comprehensive blood glucose management. Furthermore, in the management of ASCVD risk factors, management goals have been adjusted according to the most recent clinical evidence.

The guideline is based on the most recent evidence‐based medicine, to guide and help clinicians perform all‐around and full‐cycle standardized integrated management for older patients with diabetes in China and improve their clinical outcomes.

## 2 GUIDANCE FOR COMPILATIONS

In recent years, as new diabetes drugs have continued to emerge, the relevant clinical research evidence has been enriched, and the prior guidelines and consensus no longer met current clinical diagnosis and treatment needs. In 2021, by integrating the most recent evidence regarding diabetes in older people in China and other countries, and clinical practice in China, the Expert Committee compiled the first guideline for diabetes mellitus in older people in China. The release of the guideline gained widespread attention, with a read count exceeding 160,000, and was widely praised in the field.

Since the release of the previous version of the guideline, additional relevant research on older people with diabetes has been reported. Therefore, the Expert Committee organized an update of the *Guideline for the Management of Diabetes Mellitus in the Elderly in China (2021 Edition)* and compiled the *Guideline for the Management of Diabetes Mellitus in the Elderly in China (2024 Edition)*. The English literature search on which the guideline was based was performed in PubMed, Web of Science, Scopus, Ovid, Cochrane Library databases, the National Institute for Health and Care Excellence (NICE), the Registered Nurses' Association of Ontario (RNAO), the National Guideline Clearinghouse (NGC), the Scottish Intercollegiate Guidelines Network (SIGN), the Joanna Briggs Institute (JBI) Library, the British Medical Journal (BMJ) Best Practice, and other guideline libraries. The Chinese literature search included literature in the China Biology Medicine disc, China National Knowledge Infrastructure, WANFANG DATA, and VIP China Science and Technology Journal Database. The inclusion criteria for literature were as follows: (1) studies of patients 65 years or older and (2) literature types of clinical guidelines, systematic reviews, meta‐analyses, randomized control trials (RCTs), cohort studies, cross‐sectional studies, case–control studies, expert consensus, etc.

Evidence in the *Guideline for the Management of Diabetes Mellitus in the Elderly in China (2024 Edition)* is rated as level A, B, or C. Level A evidence is based on multiple RCTs or meta‐analysis. Level B evidence is based on a single RCT or multiple non‐RCTs. Level C evidence is based on only the consensus opinion of experts and/or the results of small‐scale studies, retrospective studies, or registration studies. The members of the Guideline Writing Group stated each recommendation, presented relevant evidence, and described the level of evidence to the Expert Panel members, who then confirmed the literature and evidence. All Expert Group members participating in the discussion and formulation of final opinions signed a statement on conflicts of interest. The full text was compiled in accordance with the fundamental specifications for the formulation of clinical guidelines.

## 3 CHAPTER 1: EPIDEMIOLOGY OF DIABETES AND ITS COMPLICATIONS IN OLDER PEOPLE

The prevalence of diabetes increases with age, thus exhibiting an age‐associated effect.^6^ According to data from the International Diabetes Federation in 2019, China has approximately 35.5 million patients with diabetes ≥65 years old, ranking first worldwide; these patients account for one‐quarter of all older patients with diabetes worldwide and are on the rise.^7^ A large cross‐sectional study on the mainland Chinese population published in 2020 has shown that the prevalence of diabetes is 28.8% and the prevalence of prediabetes is 47.8% between 60 and 69 years old, while the prevalence of diabetes is 31.8% and the prevalence of prediabetes is 47.6% in people over 70 years old, according to the 2018 diabetes diagnostic criteria of the American Diabetes Association. Compared with other age groups, the prevalences of diabetes and prediabetes in the age group over 60 years old are the highest, the prevalences of diabetes in elder women is higher than that in the men.^8^

No precise, high‐quality data on the incidence of diabetes complications in older people are currently available. Diabetes is associated with risks of death due to ischemic heart disease, stroke, chronic liver disease, neoplasm, female chronic urinary and reproductive system diseases, etc.^9^; moreover, the mortality among older people is significantly higher in those with diabetes rather than those without diabetes.^10^

Older people are prone to a variety of concurrent chronic diseases, and the proportion of older people with T2DM with hypertension and/or dyslipidemia is as high as 79%.^11^ When formulating the clinical diagnosis and treatment plan, clinicians should conduct comprehensive assessments to control the damage caused by multiple metabolic disorders in a safe and beneficial manner, delay diabetes progression, and substantially improve patient's quality of life.

## 4 CHAPTER 2: DIAGNOSIS, CLASSIFICATION, AND CHARACTERISTICS OF DIABETES IN OLDER PEOPLE

**Key points**

1. The World Health Organization (WHO) diagnostic criteria for diabetes (1999) are used. (A)
2. Diabetes in older people can be classified into type 1 diabetes mellitus (T1DM), T2DM, and special types of diabetes. (A)
3. Diabetes in older people has unique characteristics, including atypical symptoms, multiple complications and/or concurrent diseases, etc. (B)
4. Tumor screening is recommended for newly diagnosed older patients with diabetes. (C)

### 4.1 Diagnosis of diabetes in older people

The 1999 WHO diagnostic criteria for diabetes are used, that is, the fasting plasma glucose (FPG), random plasma glucose, or 2‐h plasma glucose (2hPG) during an oral glucose tolerance test (OGTT) are primarily used for the diagnosis of diabetes, however, the diagnosis must be confirmed by a repeated test when a patient has no typical clinical symptoms of diabetes.

The diagnostic criteria for diabetes in older people are as follows: typical diabetic symptoms (polydipsia, polyuria, polyphagia, or unexplained weight loss), plus venous random plasma glucose ≥11.1 mmol L^−1^; or venous FPG ≥7.0 mmol L^−1^; or venous 2hPG of OGTT ≥11.1 mmol L^‐1^. For patients without typical diabetes symptoms, the test should be re‐performed on another day to provide confirmation (Table 1). The WHO recommends using glycosylated hemoglobin A1c (HbA_1c_) ≥6.5% as the cut point for diagnosing diabetes.^12^ HbA_1c_ tested by laboratories in China using standardized methods and strict quality control can also be used as a diagnostic indicator for diabetes. However, in conditions such as sickle cell disease, glucose‐6‐phosphate dehydrogenase deficiency, hemodialysis, recent bleeding or transfusion, and erythropoietin therapy, the diagnosis of diabetes must be based on the measurement of venous plasma glucose levels, and HbA_1c_ cannot be used.^13^ Age is associated with HbA_1c_ levels in the Chinese population.^14^, ^15^

**TABLE 1:** Diagnostic criteria for diabetes in older people.

Diagnostic criteria	Venous plasma glucose or HbA1c level
Typical diabetic symptoms (polydipsia, polyuria, polyphagia, or unexplained weight loss) plus random plasma glucose	≥11.1 mmol L^−1^
or FPG	≥7.0 mmol L^−1^
or 2hPG of OGTT	≥11.1 mmol L^−1^
or HbA_1c_	≥6.5%
For patients without typical diabetic symptoms, another test is required for confirmation.	

*Note*: Random plasma glucose refers to blood glucose at any time of the day, regardless of the time of the last meal, and cannot be used to diagnose impaired fasting glucose or impaired glucose tolerance; fasting state refers to no intake of calories for at least 8 h; HbA_1c_ should be tested in laboratories meeting the standardized measurement requirements.

### 4.2 Classification and characteristics of diabetes in older people

Diabetes in older people refers to diabetes in individuals ≥65 years old, including those diagnosed before or after 65 years old. Older patients with diabetes are mainly T2DM, but also include T1DM, and other types of diabetes. New‐onset T1DM is rare in the older population. Most T1DM is attributed to latent autoimmune diabetes in adults or may be T1DM previously diagnosed before the age of 65 years.

In the guideline, diabetes in older people is classified into T1DM, T2DM, or specific types of diabetes (e.g., monogenic diabetes, exocrine pancreatic diseases, or drug‐ or chemical‐induced diabetes) according to the WHO 1999 etiological classification system of diabetes. The older population is a high‐risk group for malignancies, and, given the use of immune checkpoint inhibitors, immune checkpoint inhibitor–associated diabetes has been reported.^16^ Such diabetes is currently recommended to be classified as drug‐induced diabetes within the category of specific types of diabetes.

In recent years, researchers have proposed several new diabetes classification schemes, each of which has limitations. The classification of older patients with diabetes is important, but greater attention should be paid to the uniqueness of these patients. Most older patients with diabetes do not have typical clinical symptoms (i.e., polydipsia, polyuria, polyphagia, and unexplained weight loss); older patients with diabetes are prone to multiple complications and/or concurrent diseases, which may manifest first. Because diabetes is associated with a variety of malignancies—e.g., 68% of patients with pancreatic cancers have elevated blood glucose (impaired glucose tolerance or diabetes)^17^, ^18^—tumor screening is recommended for newly diagnosed older patients with diabetes.

## 5 CHAPTER 3: THREE LEVELS OF PREVENTION OF DIABETES IN THE OLDER ADULTS

### 5.1 Primary prevention

For older adults with risk factors for diabetes, the goal is to reduce the incidence of diabetes. Aging is one of the risk factors of diabetes, and the older population is susceptible to diabetes. Health education should be carried out among the older population, especially in people with prediabetes, to reduce the risk of diabetes through passing on health knowledge and improvement of lifestyle (e.g., appropriate diet and exercise, etc.). In addition, it is necessary to carry out blood glucose and HbA_1c_ screening and strengthen the ASCVD risk factors management of the older population (e.g., cessation of smoking, alcohol limitation, control of blood pressure and blood lipids, etc.) and monitor blood glucose regularly in older patients taking statin.^19^

### 5.2 Secondary prevention

For older adults who already have diabetes, the goal is to reduce the incidence of diabetes complications. Older patients with diabetes should be diagnosed as early as possible. Comprehensive complications screening and important organ function assessments should be performed at the time of diagnosis, lifestyle intervention should be guided and reasonable treatment should be carried out based on patient's condition, to reduce the incidence of complications.

### 5.3 Tertiary prevention

For older patients with complications of diabetes, the goal is to reduce patients' disability and mortality rates and improve their quality of life. For these patients, timely and effective integrated treatments and multidisciplinary management should be adopted to prevent or delay the progression of diabetes complications, lower the disability and mortality rates of older patients, and improve their quality of life.

## 6 CHAPTER 4: COMPREHENSIVE GERIATRIC ASSESSMENT OF OLDER PATIENTS WITH DIABETES

**Key points**

1. CGA is performed for older patients with diabetes through a multidisciplinary team approach. (A)
2. According to the assessment results, older patients with diabetes are divided according to health status into a good health status (Group 1), intermediate health status (Group 2), and poor health status (Group 3). (A)
3. A stratified, individualized integrated treatment, nursing, and rehabilitation strategy is developed according to patient health status. (A)

CGA is a multidisciplinary process conducted to determine the physical, functional, mental, and social problems in older people, so that a coordinated plan of care can be developed to maintain and improve their health and functional status, and maximize their quality of life. Currently, the CGA scales commonly used in China include the Comprehensive Geriatric Assessment Scale for Chinese Elderly,^20^ the Standard for Chinese Healthy Elderly Assessment Scale,^21^ and the Multidimensional Health Assessment Scale of the Elderly.^22^

The health status of older patients with diabetes varies greatly among individuals, and often accompanied by varying degrees of cognitive disorders and complex underlying diseases. Therefore, CGA should be performed through a multidisciplinary team approach, in collaboration with clinicians, dietitians, rehabilitation therapists, and nurses, to develop an individualized regimen to which each patient can adhere over the long term.

According to the above‐mentioned scales, the health status of older patients with diabetes, including the comorbidity profile, hepatic and renal function, medication profile, activities of daily living (ADL) and instrumental activities of daily living (IADL), cognitive function, mental status, nutrition profile, etc., should be assessed comprehensively to divide patients into good health (Group 1), intermediate health (Group 2), or poor health (Group 3) (Table 2). Based on the assessment results, an integrated treatment, nursing, and rehabilitation strategy is individualized for each patient.

**TABLE 2:** Comprehensive geriatric assessment of older patients with diabetes.^2^

Health status grade	Physical and functional status of elderly patients with diabetes
Good Health (Group 1)	No comorbidity or has ≤2 non‐diabetes chronic illnesses (including stroke, hypertension, stage 1–3 chronic kidney disease, osteoarthritis, etc.) and no ADL impairment and ≤1 IADL impairment
Intermediate Health (Group 2)	Three or more non‐diabetes chronic illnesses (including stroke, hypertension, stage 1–3 chronic kidney disease, osteoarthritis, etc.) and/or any one of the following: (1) mild cognitive impairment or early dementia; (2) ≥2 IADL impairments
Poor Health (Group 3)	Any one of the following: (1) one or more chronic illnesses with limited treatments and reduced life expectancy (including metastatic malignancies, lung disease requiring oxygen therapy, end‐stage renal disease requiring dialysis, and advanced heart failure); (2) moderate to severe dementia; (3) ≥2 ADL impairments; (4) residence in a long‐term nursing facility

*Note*: ADL indicates activities of daily living; IADL indicates instrumental activities of daily living. Cognitive function assessment is conducted with the Mini‐Mental State Examination (MMSE) and the Montreal Cognitive Assessment (MoCA). The MMSE focuses on screening for overall cognitive function (temporal and spatial orientation, arithmetic skills, memory, language abilities, attention, and visuospatial skills), and test scores are closely associated with the patient's education level. Mocap is a rapid screening tool used for the detection of mild cognitive impairment, encompassing 11 tasks within eight cognitive domains: attention and concentration, executive functions, memory, language, visuospatial structure skills, abstract thinking, calculation, and orientation.

Appendices A and B describe the ADL and IADL assessment methods.^23^, ^24^

## 7 CHAPTER 5: HEALTH EDUCATION FOR OLDER PATIENTS WITH DIABETES

**Key points**

1. The health education should be individualized according to the characteristics of each older patient with diabetes. (A)
2. The contents of education on older patients with diabetes cover the etiology, harms, treatment, and treatment goals of diabetes. (B)

At the early stage of disease diagnosis, health care professionals (HCPs) and family members need to help patients face up to the disease; enable them to receive diabetes education and understand diabetes‐related knowledge; alleviate their fears, self‐defeating thoughts, and other negative thoughts. This conducive behavior should be affirmed in a timely manner to encourage the patient's diabetes management. Guide them to have a positive self‐evaluation and make them accept and actively participate in the management of diabetes throughout the process.

Older patients with diabetes usually have a relatively long duration of disease and multiple complications and comorbidities; as a result, individualized health education should be carried out according to the characteristics of each patient. Older patients need to be assessed before health education is carried out, including basic information, education level, economic status, previous treatment status, blood glucose level, comorbidities, cognitive function, and the presence of caregivers. The contents of education should cover the etiology, disease progression and clinical manifestations of diabetes, hazards of diabetes, identification and treatment of acute and chronic complications of diabetes, goal of individualized treatment, lifestyle intervention, characteristics of various kinds of drugs, choice and use of clinical drugs, and how to carry out blood glucose monitoring, etc. Health education on patients themselves, family members and caregivers, and community‐related personnel should be strengthened to enable them to have a correct understanding of disease‐related knowledge and to avoid excessively aggressive or relaxed blood glucose management, thus improving the quality of life of older patients with diabetes. Diabetes education can be carried out in the forms of collective education and more targeted community group education, peer education, and individual education. If possible, remote education via WeChat public account, mobile app, and online training course, etc., can also be adopted. These methods can be carried out simultaneously to complement each other, to better pass on the necessary information to patients. In recent years, there have been numerous studies exploring different educational approaches for older patients with diabetes, such as the spousal‐assisted management model and the PRECEDE‐PROCEED model.^25^, ^26^ Effective educational approaches will contribute to the comprehensive management of older patients with diabetes. With the rapid development of artificial intelligence technology, recent years, there have also been several studies investigating the role of artificial intelligence in diabetes education.^27^

## 8 CHAPTER 6: GLYCEMIC GOALS FOR OLDER PATIENTS WITH DIABETES

**Key points**

1. Consideration should be given to the benefit‐to‐risk ratio in setting glycemic goals for older patients with diabetes. (A)
2. Stratified goals should be set for glycemic control according to the health status of older patients. (B)
3. HbA_1c_ and point glucose levels can be used as indicators for the assessment of glycemic control in older patients with diabetes. (A)
4. Glucose variability should be monitored; when necessary, indexes of glucose variability can be used as supplementary indicators for glycemic goals. (C)

Strict control of blood glucose in older patients with diabetes provides limited benefits and somewhat increases the risk of hypoglycemia, which is extremely harmful to older patients and should be avoided to the greatest extent possible. Therefore, assessing treatment plans by considering the benefit‐to‐risk ratio is critical. Stratifying older patients and setting individualized glycemic goals are important. For older patients with diabetes with poor health status (Group 3), glycemic goals can be relaxed to some extent. However, the following consequences of less stringent blood glucose control must be avoided: (1) clear symptoms of diabetes; (2) elevated risk of infection; and (3) hyperglycemic crisis.

Based on the overall health assessment of older patients with diabetes, depending on whether they use drugs that increase the risk of hypoglycemia, the recommended glycemic goals are shown in Table 3. For older patients taking drugs that increase the risk of hypoglycemia, the HbA_1c_ goal should not be too low. Therefore, a lower limit of the glycemic goal should be clearly set for these patients, to mitigate the risk of hypoglycemia. Glucose indicators such as the time in range (TIR), time below range (TBR), time above range (TAR), and coefficient of variation (CV) can reveal glucose variability. According to international consensus, these indicators can be used as supplementary glycemic goals^28^ (Table 4). Regarding postprandial glucose control, no adequate clinical evidence or guidelines are available for setting goals. One method to determine the postprandial glycemic goal is based on the average postprandial glucose of a given HbA_1c_ level (Standards of Medical Care in Diabetes^29^): HbA_1c_ of 6.50%–6.99% corresponds to blood glucose of 9.1 mmol L^−1^; HbA_1c_ of 7.00%–7.49% corresponds to blood glucose of 9.8 mmol L^−1^; HbA_1c_ of 7.50%–7.99% corresponds to blood glucose 10.5 mmol L^−1^; and HbA_1c_ of 8.00%–8.50% corresponds to blood glucose of 11.4 mmol L^−1^.

**TABLE 3:** Glycemic goals for older patients with diabetes.

Blood glucose monitoring index	Not taking hypoglycemia‐inducing drugs			Taking hypoglycemia‐inducing drugs		
	Good health status (Group 1)	Intermediate health status (Group 2)	Poor health status (Group 3)	Good health status (Group 1)	Intermediate health status (Group 2)	Poor health status (Group 3)
HbA_1c_ (%)	<7.5	<8.0	<8.5	7.0–7.5	7.5–8.0	8.0–8.5
Fasting or pre‐prandial glucose level (mmol L^−1^)	5.0–7.2	5.0–8.3	5.6–10.0	5.0–8.3	5.6–8.3	5.6–10.0
Bedtime glucose level (mmol L^−1^)	5.0–8.3	5.6–10.0	6.1–11.1	5.6–10.0	8.3–10.0	8.3–13.9

*Note*: HbA_1c_ indicates glycated hemoglobin; examples of hypoglycemia‐inducing drugs include insulin, sulfonylureas, meglitinides, etc.; the HbA_1c_, fasting, or pre‐prandial blood glucose and bedtime glycemic goals are derived from the Treatment of Diabetes in Older Adults Guideline Resources issued by the American Endocrine Society.^2^

**TABLE 4:** Glucose variability control goals for older patients with diabetes.

Glucose variation indicator	Blood glucose range	Control goal	
		Proportion of day	Duration per day
TIR	3.9–10.0	>50%	>12 h
TBR	<3.9	<1%	<15 min
TAR	>13.9	<10%	<144 min
CV	Not applicable	≤36%	

Abbreviations: CV, coefficient of variation; TAR, time above range; TBR, time below range; TIR, time in range.

## 9 CHAPTER 7: LIFESTYLE INTERVENTION OF OLDER PATIENTS WITH DIABETES

**Key points**

1. Lifestyle intervention is the fundamental treatment for older patients with diabetes; all older patients with diabetes should receive lifestyle intervention. (A)
2. Provide individualized lifestyle guidance according to the health status level of older patients with diabetes. (B)
3. Assess the nutritional status of older patients with diabetes and identify malnutrition as early as possible; pay attention to appropriately increasing the protein and energy intake when developing the nutritional treatment regimen. (A)
4. Exercise risk and physical fitness should be assessed for older patients with diabetes before starting their exercise therapy. (A)
5. Encourage older patients to choose appropriate exercises that that can be sustained over the long term (e.g., aerobic exercise, resistance exercise, etc.), prevent older patients from falling during the exercise, be vigilant about hypoglycemia during and after exercise, and give timely treatment once it occurs. (B)

Lifestyle intervention is the fundamental treatment of older patients with diabetes, all of whom should receive lifestyle therapy. For some older patients with diabetes in good health (Group 1) with mildly increased blood glucose levels, their targeted glucose can be achieved by only lifestyle intervention.

### 9.1 Medical nutrition therapy

Medical nutrition therapy (MNT) is fundamental and should be applied through the whole course of diabetes mellitus management. It plays a crucial role in achieving the goals of blood glucose, blood pressure, and lipids; maintaining a healthy weight; and preventing or delaying complications associated with diabetes. First, the nutritional status of older patients with diabetes should be assessed. The Nutrition Risk Screening 2002 (NRS‐2002), Short‐Form Mini Nutritional Assessment (MNA‐SF), and other malnutrition screening tools should be adopted periodically to identify patient's nutrition risk. Malnutrition in older patients may cause a series of issues such as prolonged length of hospital stay, increased medical expenses, and increased re‐hospitalization rate, etc. Therefore, early identification and management of malnutrition can help prevent and delay the occurrence and progression of complications.

It is difficult for the elderly to change their diet habits. It is possible to make appropriate adjustments to develop individualized nutritional therapy plans based on their dietary habits, considering factors such as the degree of willingness to modify dietary structure and the ability to obtain healthy food, and considering metabolic control goals, total calorie intake, and nutritional quality. MNT should be coordinated with the patient's overall lifestyle, including their exercise status and medication use. Because muscle mass in older patients are tend to be low and a sufficient energy intake can avoid muscle proteolysis, the protein intake, predominantly the intake of high‐quality protein rich in branched‐chain amino acids such as leucine, should be increased as appropriate.^30^ Healthy older people need to ingest 1.0–1.3 g kg^−1^ d^−1^ of protein, those with concurrent acute/chronic diseases need to ingest 1.2–1.5 g kg^−1^ d^−1^ of protein, and those with concurrent sarcopenia or severe malnutrition need to ingest at least 1.5 g kg^−1^ d^−1^ of protein.^31^ In addition to animal protein, plants with high‐quality protein are also acceptable.^32^ Carbohydrates are the main energy source of older patients with diabetes in China, and monitoring carbohydrate intake is an important strategy for achieving blood glucose goals, but there is still no consensus on the optimal amount of carbohydrate intake for older patients with diabetes. Intake of foods rich in dietary fiber while consuming carbohydrates can delay blood glucose elevation, reduce glycemic variability, and improve lipid control. As dietary fiber increases the feeling of fullness and delays gastric emptying, older patients with gastroparesis and functional gastrointestinal disorders should avoid excessive intake. Attention should be paid to patients' ingestion sequence of carbohydrate, protein, and vegetables, since later ingestion of carbohydrates can reduce the speed of increase in patient's postprandial plasma glucose level.^33^ For older diabetes mellitus patients with long‐term imbalanced diet, attention should also be paid to supplementation of vitamins and minerals. Compared to the non‐diabetic population, older patients with diabetes are at higher risk of malnutrition and more likely to develop sarcopenia and frailty.^34^ As a result, these patients should avoid excessive limitation of energy intake, pay attention to rational diet and balanced nutrition, and be alert to malnutrition.

### 9.2 Exercise therapy

Exercise is one of the most effective methods for preventing and managing older people with diabetes,^35^, ^36^ and lifestyle intervention based on regular exercise can improve the insulin sensitivity of patients with diabetes.^37^ However, older patients with diabetes often suffer from multiple concurrent chronic diseases, for example, osteoarthropathy, which causes a decline in their walking ability, and cerebrovascular disease, peripheral neuropathy, or severe sarcopenia, which make patients are prone to fall. Therefore, it is necessary to carry out exercise risk evaluation for older patients with diabetes before exercise therapy, which is based on patients' medical history, family history, physical activity ability, and related medical examination results. The physical ability of older patients should be assessed by multiple tests including cardio‐respiratory endurance, body composition, muscle strength and muscular endurance, flexibility, and balance ability, etc., to provide a basis for the development of the exercise therapy plans. In addition, older patients often need to take a variety of medicines, thus they should be instructed to reasonably arrange the interval between medication and exercise, and the influence of exercise on drug metabolism should be assessed to avoid the occurrence of exercise‐related events such as hypoglycemia and hypotension. Hypoglycemia may occur during exercise, or after exercise as delayed hypoglycemia; thus it is necessary to strengthen blood glucose monitoring before, after, and during exercise and to pay attention to whether the patient has hypoglycemic symptoms such as dizziness, palpitations, fatigue, trembling hands, or sweating. Once it occurs, stop exercising immediately and deal with it in a timely manner. Patients with concurrent cardiac disorders should follow the exercise guidance program for patients with cardiac disorders.

Moderate‐intensity aerobic exercise is preferred for older patients with diabetes, while low‐intensity aerobic exercise is an option for those with poor exercise ability. Low‐ and moderate‐intensity aerobic exercises are safe for most older patients with diabetes, including brisk walking, fitness dance, rhythmic gymnastics, cycling, underwater exercise, and jogging. The intensity of exercise can be evaluated through the patient's feeling of fatigue, which is usually manifested as feeling of rapid heartbeat, slight sweat, and feeling of slight fatigue during moderate‐intensity exercise, or being able to speak in complete sentences but unable to sing during exercise. It is suggested to exercise 5 to 7 days each week, preferably every day. The best time for exercise is 1 hour after meals, and it is suggested to exercise approximately 20 min after each meal. Exercise before meals should be started after appropriate ingestion of carbohydrates according to the blood glucose level.

Resistance training is also suitable for older adults. The process of exerting muscle force against resistance to produce movement is called resistance training. Resistance training can improve the strength, bone density, lean body mass, insulin sensitivity and blood pressure, HbA_1c_, and lipid control of older people with T2DM.^38^, ^39^ Resistance exercise by dumbbell, resistance band, and other instruments or using the patient's self‐weight (e.g., push‐ups, burpees, squats, leg raises, biceps curls, calf raises) is also suitable for the elderly population. It is suggested to exercise 2 to 3 times a week, perform 1 to 3 groups of exercises each time, and repeat each group/action 10 to 15 times.

Older patients with diabetes often suffer from problems such as declining balance ability. Strengthening flexibility and balance can enhance the body's coordination and balance capabilities, thus reducing the risk of falls in older patients with diabetes^40^ and increasing exercise compliance. Alternating single‐legged standing and walking in a straight line are effective ways to enhance balance. Tai chi, Baduanjin, Wuqinxi, and yoga can also improve coordination and balance. A meta‐analysis found that tai chi has a positive effect on improving the ability of single‐legged standing in elderly patients with T2DM and improving blood glucose.^41^ Balance training is recommended at least 2 to 3 times a week.

Aerobic exercise, resistance training, and balance exercises all have varying benefits for older patients with diabetes. Based on the evidence, it is recommended in most consensus or guidelines that older people engage in various modalities of exercise, including aerobic, resistance, flexibility, and balance training, to improve health status through a combination of structured exercise prescription and random activities.^42^, ^43^ Older patients with diabetes can increase physical activities in daily life according to their own conditions, such as low‐intensity housework, yard activities, etc., and reduce sitting time. They should get up and move for 1–5 min every 30 min of sitting.

## 10 CHAPTER 8: GLYCEMIC MEDICATIONS AND TREATMENT STRATEGIES FOR OLDER PATIENTS WITH TYPE 2 DIABETES

**Key points**

1. Both health status and glycemic goal should be considered for older patients with T2DM, to formulate glycemic therapies. (B)
2. Highly individualized glycemic therapies should be prioritized, by considering factors such as organ function (e.g., heart, liver, and kidneys), complications, comorbidities, risk of hypoglycemia, frailty, weight, and the preferences of patients and their families. (A)
3. Lifestyle intervention is the cornerstone of treating older patients with T2DM; medications are introduced if glycemic goals are not achieved through lifestyle changes alone. (A)
4. Safe and convenient glycemic therapies should be preferentially chosen for older patients with T2DM. (A)
5. Older T2DM patients with ASCVD or at high risk of ASCVD should preferentially receive sodium‐glucose cotransporter 2 inhibitors (SGLT2 inhibitors) or glucagon‐like peptide‐1 receptor agonists (GLP‐1RAs) with evidence of ASCVD benefits. (A)
6. For older patients with T2DM with heart failure or chronic kidney disease (CKD), SGLT2 inhibitors should be prioritized. For older patients with T2DM and CKD who are intolerant of SGLT2 inhibitors, GLP‐1RAs with evidence of CKD benefits should be considered. (A)
7. De‐intensification of insulin therapy should be emphasized for older patients with T2DM. (B).

Considering the comprehensive assessment of each patient's health and corresponding glycemic goals, older patients with T2DM whose blood glucose levels are uncontrolled after lifestyle interventions should initiate medication therapy as early as possible. The principles of medication treatment include (1) prioritizing the use of medications with a relatively low risk of hypoglycemia; (2) selecting convenient and highly compliant medications, and decreasing the risk of polypharmacy; (3) balancing the benefit‐to‐risk ratio to avoid overtreatment; (4) monitoring factors such as liver and kidney function, cardiovascular health, complications, and comorbidities; and (5) recommending against high‐risk medications with hypoglycemia and noticeable weight loss in frail older patients.

### 10.1 Metformin

Metformin is one of the first‐line glycemic drugs recommended for older patients with T2DM in various international and Chinese guidelines and/or consensus statements. Metformin is a Grade 1 recommended glycemic drug in older patients with T2DM in the guideline. The decisive factor for metformin use and dosage decreases is the estimated glomerular filtration rate (eGFR). Gastrointestinal adverse events and weight loss are contraindications for metformin use in some older patients. For older patients, starting with a low dose (500 mg d^−1^) and escalating the dose gradually, to a maximum dose not exceeding 2550 mg d^−1^, is recommended. Sustained‐release or enteric‐coated formulations may alleviate gastrointestinal reactions, and slow‐release formulations require less frequent administration.^44^ If older patients already have impaired kidney function, regular monitoring of kidney function is warranted, and the metformin dosage should be adjusted accordingly.^45^ For older patients with an eGFR of 45–59 mL min^−1^ (1.73 m^2^)^−1^, dosage decreases should be considered, and when the eGFR is <45 mL min^−1^ (1.73 m^2^)^−1^, discontinuation should be considered.^45^ Metformin is contraindicated in older patients with severe infections, trauma, and conditions causing tissue hypoxia (such as decompensated heart failure or respiratory failure). For patients with eGFR ≥60 mL min^−1^ (1.73 m^2^)^−1^ undergoing examinations with a contrast agent with iodine, metformin should be discontinued on the day of the examination and can be resumed at least 48 h after the re‐examination, provided that kidney function has not worsened. If the patient's eGFR is 45–59 mL min^−1^ (1.73 m^2^)^−1^, metformin should be stopped 48 h before administration of iodine contrast agents or general anesthesia. The discontinuation should last for another 48–72 h, and resumption is allowed after kidney function has been confirmed not to have worsened in the re‐examination.^45^ Metformin increases the risk of vitamin B_12_ deficiency in older people with diabetes^46^; therefore, vitamin B_12_ levels should be monitored regularly during therapy.

### 10.2 Sulfonylureas

The commonly used sulfonylureas include glibenclamide, gliclazide, glipizide, gliquidone, and glimepiride. Although sulfonylureas have a noticeable hypoglycemic effect, they carry risks of hypoglycemia and weight gain. Long‐acting sulfonylureas tend to exhibit the above adverse effects more frequently, particularly in older people, thus necessitating cautious use.^47^ Short‐acting formulations, sustained‐release formulations, and controlled‐release preparations with consistent and stable concentrations are preferred after careful evaluation of their benefits and risks. Sulfonylureas are a Grade 3 glycemic recommendation for older patients with T2DM. When co‐administered with drugs that are cleared by hepatic P450 enzymes such as CYP2C9 and CYP2C19 (e.g., statins, antibiotics, selected cardiovascular agents, and proton pump inhibitors), vigilance is imperative to avoid hypoglycemic events^48^ and other adverse events. Gliquidone, with a plasma half‐life of 1.5 h and only 5% of its metabolites cleared via the kidneys, is a preferred sulfonylurea choice for older patients with T2DM with mild to moderate renal impairment.

### 10.3 Glinides

The main glinides are repaglinide and nateglinide. The hypoglycemic effect and the risk of weight gain of glinides are similar to that of sulfonylureas, while the risk of hypoglycemia is lower.^49^ These drugs are Grade 2 recommended glycemic drugs for older patients with T2DM. Glinides should be taken within 15 min before meals, thus requiring high patient compliance. Glinides are cleared primarily by the liver. For older patients with mild to moderate kidney impairment, no dosage adjustment is needed when nateglinide is used. For older patients with impaired kidney function, the starting dose of repaglinide also need not be changed.

### 10.4 Alpha‐glucosidase inhibitors

Alpha‐glucosidase inhibitors, primarily acarbose, voglibose, and miglitol, function by inhibiting the activity of alpha‐glucosidase in the small intestine, thus slowing the degradation and absorption of carbohydrates, and consequently decreasing postprandial blood glucose. They are suitable for patients with diabetes who have carbohydrate‐rich diets and experience elevated blood glucose after meals. Common adverse reactions to this class of drugs include abdominal bloating, diarrhea, and increased flatus, which have somewhat limited their use in older population.^50^ Alpha‐glucosidase inhibitors are classified as a Grade 2 glycemic recommendation for older patients with T2DM. Starting with a low dose and gradually escalating the dose is advised. When these drugs are used alone, the risk of hypoglycemia is relatively low. In the event of hypoglycemia, glucose should be administered, because carbohydrates such as starch have limited efficacy in raising blood glucose.

### 10.5 Thiazolidinediones

Thiazolidinediones (TZDs), such as rosiglitazone and pioglitazone, are insulin sensitizers that exert glycemic effects by enhancing the sensitivity of insulin in skeletal muscle, liver, and adipose tissues. When used independently, these drugs generally do not induce hypoglycemia, but when they are combined with insulin or insulin secretagogues, the risk of hypoglycemia is elevated.^49^ TZDs are classified as a Grade 3 glycemic recommendation for older patients with T2DM. Studies have indicated that pioglitazone can reduce the composite endpoint risk of all‐cause mortality rate, non‐fatal myocardial infarction, and stroke in T2DM patients with high risk of macrovascular complications.^51^ A meta‐analysis has revealed that pioglitazone may decrease the risk of recurrent stroke in patients who have experienced prior ischemic stroke or transient ischemic attack with insulin resistance, prediabetes, or diabetes.^52^ Retrospective cohort studies have suggested that pioglitazone decreases the risk of major cardiovascular events and dementia in older patients with T2DM.^53^ Thus, TZDs can be a choice for older patients with T2DM with severe insulin resistance. However, TZDs may increase the risk of weight gain, edema, fractures, and heart failure.^54^ Caution is recommended when these drugs are used in older patients with T2DM who are receiving insulin or who have congestive HF, osteoporosis, or risks of falls or fractures. Combining low‐dose TZDs with other glycemic medications may help attenuate adverse reactions.^47^, ^55^, ^56^

### 10.6 Dipeptidyl peptidase IV inhibitors

Dipeptidyl peptidase IV inhibitors (DPP‐4 inhibitors), such as sitagliptin, vildagliptin, saxagliptin, alogliptin, linagliptin, trelagliptin, retagliptin, and teneligliptin, are among the Grade 1 glycemic treatments recommended for older patients with T2DM in recent Chinese and international guidelines and/or consensus statements. These drugs inhibit the activity of the DPP‐4 enzyme, thus increasing endogenous glucagon‐like peptide‐1 (GLP‐1) levels. GLP‐1 facilitates glucose concentration–dependent endogenous insulin secretion while suppressing glucagon secretion, thereby lowering blood glucose levels.^57^ Currently most approved DPP‐4 inhibitors on the Chinese market are administered daily (or twice daily for vildagliptin), except for trelagliptin, which is administered weekly. When used alone, these drugs generally do not induce hypoglycemia. DPP‐4 inhibitors have neutral effects on body weight and cause few gastrointestinal reactions^58^ and therefore are suitable treatment choice for older patients.^59^ In older patients already receiving stable basal insulin therapy, addition of linagliptin has been demonstrated to enhance glycemic control and help older patients achieve their goals safely.^60^ DPP‐4 inhibitors are categorized as Grade 1 recommended glycemic drugs for older patients with T2DM.

The results from cardiovascular outcome trials (CVOTs) for sitagliptin, linagliptin, and saxagliptin in older patient subgroups have indicated that these drugs do not increase the risk of major adverse cardiovascular events (MACEs), either 3P or 4P, in older patients.^61^, ^62^, ^63^ Linagliptin specifically does not elevate the risk of renal composite outcomes, defined as death from kidney disease, progression to terminal kidney disease, or a sustained eGFR decline ≥40%, in older patients.^62^ However, saxagliptin has been associated with an elevated risk of heart failure hospitalization.^63^

No dose adjustment is warranted when linagliptin is administered in patients with hepatic insufficiency or saxagliptin is administered in patients with liver impairment; sitagliptin does not require dosage adjustment in patients with mild to moderate hepatic insufficiency. However, caution should be exercised with alogliptin in patients with liver disease, and vildagliptin is contraindicated for patients with abnormal liver function (serum alanine aminotransferase or aspartate transaminase levels exceeding three times the upper limit of normal or increasing continuously). Linagliptin and teneligliptin can be used in older patients with any level of kidney function without dosage adjustment. For the other DPP‐4 inhibitors, dosage adjustment or discontinuation may be necessary, according to kidney function. If pancreatitis is suspected in a patient, the use of DPP‐4 inhibitors should be terminated.

### 10.7 Sodium‐glucose cotransporter 2 inhibitors

Sodium‐glucose cotransporter 2 inhibitors (SGLT2 inhibitors) increase urinary glucose excretion by inhibiting the activity of SGLT2 in the proximal renal tubules, thereby lowering glucose levels.^64^ SGLT2 inhibitors currently approved for clinical use in China include dapagliflozin, empagliflozin, canagliflozin, ertugliflozin, and henagliflozin. These drugs are effective and safe in older patients.^65^ Because the mechanism of these drugs does not rely on insulin for decreasing blood glucose,^66^ hypoglycemia rarely occurs.^67^, ^68^, ^69^, ^70^ SGLT2 inhibitors also play roles in weight loss, particularly in decreasing visceral fat.^71^ The EMPA‐ELDERLY study has demonstrated that empagliflozin decreases body weight without compromising muscle mass and body strength in older patients.^72^

SGLT2 inhibitors have demonstrated cardiovascular and renal benefits. A meta‐analysis has indicated that SGLT2 inhibitors consistently decrease the risk of 3P‐MACE, heart failure hospitalization, and renal outcomes in older patients and other age groups.^73^ SGLT2 inhibitors are Grade 1 glycemic drugs for older patients with T2DM and are recommended as the first choice for older patients with established/high risk of ASCVD, HF, and CKD. CVOTs have indicated that empagliflozin and canagliflozin decrease the risk of 3P‐MACE in patients with T2DM and have demonstrated consistent results in both the older subgroup and the general population.^74^, ^75^ CVOTs also have suggested that dapagliflozin and empagliflozin consistently decrease the risk of hospitalization for heart failure in patients with T2DM, in both the older subgroup and the general population.^74^, ^76^ Renal outcome trials (ROTs) have shown indicated that canagliflozin is associated with diminished risk of cardiovascular death or heart failure hospitalization^77^ in both the older patient subgroup and the general population. CVOTs have indicated that dapagliflozin and empagliflozin improve renal outcomes,^78^, ^79^ achieving improvements in composite cardio‐renal outcomes with dapagliflozin and renal outcomes with empagliflozin in both the older subgroup and the general population. A ROT of canagliflozin has confirmed its role in improving renal outcomes and has demonstrated consistent results in the older subgroup and the general population.^74^, ^75^, ^76^ The EMPA‐KIDNEY study has concluded that empagliflozin decreases the risk of renal disease progression or cardiovascular death in patients with CKD (with or without T2DM).^80^ Moreover, the DAPA‐CKD study has suggested that dapagliflozin decreases the risk of worsening renal function, terminal‐stage renal disease, and death from renal or cardiovascular diseases in patients with CKD (with or without T2DM) and has indicated similar effects in the older subgroup and the general population.^81^, ^82^ The EMPEROR‐Reduced and EMPEROR‐Preserved studies have shown that empagliflozin decreases the composite endpoint risk of cardiovascular death and heart failure hospitalization in patients with HF at any ejection fraction (with or without T2DM) and have observed similar effects in the older subgroup and the general population.^83^, ^84^, ^85^ The DAPA‐HF and DELIVER studies have demonstrated dapagliflozin's efficacy in avoiding the composite endpoint of cardiovascular death and hospitalization or emergency care due to heart failure in patients with HF at any ejection fraction (with or without T2DM), with comparable results in the older subgroup and the general population.^86^, ^87^, ^88^ The CVOT study has concluded that ertugliflozin decreases the risk of heart failure hospitalization without increasing the risk of 3P‐MACE and cardiovascular death and has indicated consistent results in the older subgroup and the general population.^89^ For henagliflozin, studies on its efficacy regarding cardiac function or urinary albumin in patients with T2DM are ongoing.

Common adverse reactions of SGLT2 inhibitors include genitourinary system infection and hypovolemia. Reports of ketoacidosis have also been observed in post‐marketing clinical surveillance, and the risk may be elevated in older patients.^90^ In addition, SGLT2 inhibitors lower blood pressure and promote weight loss. Therefore, special attention should be paid to monitoring patient blood pressure and avoiding hypotension. Caution should be exercised when these drugs are administered to frail patients. If the eGFR is <45 mL min^−1^ (1.73 m^2^)^−1^, canagliflozin and ertugliflozin are not recommended. If the eGFR is <30 mL min^−1^ (1.73 m^2^)^−1^, henagliflozin is not recommended, and canagliflozin and ertugliflozin are prohibited. If the eGFR is <25 mL min^−1^ (1.73 m^2^)^−1^, dapagliflozin should not be initiated, whereas for an eGFR <20 mL min^−1^ (1.73 m^2^)^−1^, empagliflozin should not be initiated. However, if empagliflozin or dapagliflozin has already been used, the treatment should ideally not be discontinued, unless patients are intolerant or require renal replacement therapy.^91^ These drugs decrease glucose levels by increasing urinary glucose excretion; therefore, for lowering blood glucose, dapagliflozin is not recommended when the eGFR is <45 mL min^−1^ (1.73 m^2^)^−1^, and empagliflozin is not recommended when the eGFR is <30 mL min^−1^ (1.73 m^2^)^−1^.

### 10.8 Glucagon‐like peptide‐1 receptor agonists

GLP‐1RAs bind the GLP‐1 receptor, thereby promoting insulin secretion and inhibiting glucagon secretion, and decreasing blood glucose, in a glucose concentration–dependent manner. These drugs slow gastric emptying, inhibit the appetite center, and decrease eating. Furthermore, they play roles in decreasing body weight, lowering blood pressure, and regulating lipids.^92^, ^93^, ^94^ The risk of hypoglycemia is low when GLP‐1RAs are used alone. The safety and efficacy of GLP‐1RAs in older people (>65 years) are similar to those in all adults.^95^, ^96^, ^97^ The GLP‐1RAs currently approved in China include exenatide, liraglutide, lixisenatide, dulaglutide, beinaglutide, loxenatide, and semaglutide, all of which are subcutaneous injection preparations. Liraglutide is administered once per day at any time. Lixisenatide is administered once per day before any meal. Exenatide weekly formulation, loxenatide, dulaglutide, and semaglutide are administered once per week at any time. The flexible administration of GLP‐1RAs supports compliance among older patients with diabetes, and weekly formulations are further increasing compliance.

Some GLP‐1RAs have proven cardiovascular benefits. A meta‐analysis has indicated that these drugs significantly decrease the risk of 3P‐MACE, cardiovascular death, and stroke, and their effects are consistent in both older people and non‐older population.^73^ GLP‐1RAs also decrease the risk of adverse renal outcomes, primarily through decreasing urinary albumin excretion.^98^ GLP‐1RAs are recommended as first‐choice drugs for older patients with established/high risk of ASCVD. They can serve as an alternative treatment for older patients with CKD who cannot tolerate SGLT2 inhibitors. GLP‐1RAs are generally Grade 2 glycemic drugs for older patients but are among the Grade 1 recommendations for patients with established/high risk of ASCVD. Dulaglutide significantly reduces 3P‐MACE for T2DM patients with cardiovascular disease or at high risk.^99^ Dulaglutide has shown evidence of primary prevention in T2DM patients with high risk of ASCVD; and the results from baseline heterogeneity analysis are consistent in patients aged ≥66 years and <66 years.^99^ Liraglutide significantly reduces the risk of 3P‐MACE in T2DM patients complicated with high risk of cardiovascular diseases.^100^ Semaglutide significantly decreases the risk of 3P‐MACE in patients with T2DM with cardiovascular disease, chronic HF, CKD, age >60 years, or high cardiovascular risk factors. In the baseline heterogeneity analysis, the results were consistent for patients ≥65 years and <65 years of age.^101^ CVOTs of lixisenatide and exenatide have demonstrated no increased risk of MACE.^102^, ^103^ CVOT secondary endpoints and exploratory analysis of renal outcomes of exenatide, liraglutide, lixisenatide, dulaglutide, and semaglutide have suggested that these therapies decrease the risk of renal composite outcomes and depressed urinary albumin excretion in patients with T2DM.^99^, ^100^, ^101^, ^102^, ^103^

Studies have explored the clinical benefits of combining SGLT2 inhibitors and GLP‐1RAs. A meta‐analysis has suggested that, compared with single drug treatment, the combination of two classes of drugs confers more benefits regarding blood glucose, blood pressure, and blood lipid.^104^ A retrospective cohort study has found that, compared with single drug treatment, combined therapy with SGLT2 inhibitors and GLP‐1RAs achieves better all‐cause mortality and cardiovascular benefits.^105^ However, little high‐quality evidence to date has demonstrated the clinical benefits of combined therapy with these two drugs in the older population.

GLP‐1RAs that are easy to use and provide extra benefits other than glucose lowering are recommended.^106^ The main adverse reactions of GLP‐1RAs are nausea, vomiting, diarrhea, decreased appetite, other gastrointestinal adverse events, and slowed gastric emptying. Vigilance is necessary to avoid inducing or aggravating malnutrition, sarcopenia, or frailty in older patients with T2DM. In addition, some studies have observed an increased risk of adverse reactions such as intestinal obstruction, gastroparesis, and pancreatitis after use of GLP‐1RAs.^107^ Therefore, for older patients, it is necessary to assess their potential risks and benefits.

### 10.9 Insulin

If lifestyle interventions and non‐insulin treatments fail to achieve glycemic goals in older patients with T2DM, insulin therapy may be introduced. Before initiation of insulin therapy in older patients, factors such as the overall health, characteristics of elevated blood glucose, and risk of hypoglycemia must be adequately considered. The benefit‐to‐risk ratio should be assessed for each individual to formulate customized therapies.

For the initial insulin treatment, the preferred choices are basal insulin, biphasic insulin, or a fixed combination of basal insulin and GLP‐1RAs, because their ease of administration and high compliance make them suitable for most older patients.^47^ The preferred choice of basal insulin should be a dosage form that maintains a stable concentration in the blood (e.g., Insulin Glargine U100, Insulin Degludec, and Insulin Glargine U300). Basal insulin should be administered in the morning to decrease the risk of hypoglycemia, particularly nocturnal hypoglycemia. The starting dose may be calculated according to the body weight and is usually set at 0.1–0.3 U kg^−1^ d^−1^.^108^ For patients with HbA_1c_ >8.0%, a dose of 0.2–0.3 U kg^−1^ d^−1^ may be used as the starting basal dose.^109^ The dosage is adjusted every 3 to 5 days depending on the fasting blood glucose level until the goal is reached. If the fasting blood glucose goal is reached, but the goal for HbA_1c_ has not been reached, focus should be placed on postprandial blood glucose, and prandial insulin may be introduced if necessary.^2^ Basal insulin combined with prandial insulin (three times per day) mimics the human body's physiological insulin secretion pattern, but the complicated administration regimen may compromise patients' compliance with long‐term therapy. In addition, this regimen is not suitable for older patients with poor health status (Group 3) or with short life expectancy. Biphasic insulin injection once or twice per day is as effective as multiple injections but requires fewer administration doses, thus supporting patient compliance.^110^ In addition, its pharmacokinetics, effectiveness, and safety profiles are similar between older patients with diabetes and other patients.^111^, ^112^, ^113^ Premixed insulin requires less frequent administration than a basal‐prandial combination. However, in older patients, particularly those with long disease duration, poor pancreatic islet function, or irregular meals, premixed human insulin or premixed insulin analogues may increase the risk of hypoglycemia.^114^

If older patients with diabetes have HbA_1c_ >10.0% or symptoms of hyperglycemia (e.g., polydipsia or polyuria), or show evidence of catabolism (e.g., weight loss) or severe hyperglycemia (fasting blood glucose >16.7 mmol L^−1^), short‐term insulin therapy may be applicable, depending on patients health status and treatment goals. Except for patients with pancreatic islet failure, older patients with diabetes should promptly decrease the frequency of insulin injections, and their antidiabetic therapy should be optimized after short‐term insulin therapy has stabilized the blood glucose and addressed the high glucose toxicity.^115^

In older patients with diabetes, the strategy for insulin therapy should involve de‐intensification. For patients already receiving insulin, assessments should be conducted to determine whether insulin therapy is necessary and whether the regimen can be de‐intensified. Multiple injections of insulin are not recommended for older patients with diabetes who are senile or have short life expectancy or poor health status (Group 3). Compared with multi‐injection insulin therapy, a fixed combination of basal insulin and GLP‐1RAs, biphasic insulin, and basal insulin combined with oral glycemic drugs require fewer administration injections and thus enables a simplified regimen. Older patients with diabetes who can achieve glycemic goal with non‐insulin therapy should gradually decrease their insulin dose until insulin is discontinued. Otherwise, patients relying on insulin for blood glucose control should receive the simplest possible insulin therapy, with the following considerations: (1) Minimize the frequency of injections; (2) Use long‐acting or ultra‐long‐acting insulin analogues to regulate fasting and pre‐prandial blood glucose to a satisfactory level. If postprandial glucose goals are not met, mealtime insulin may be considered. If patients have no contraindications, switching to a fixed combination of basal insulin and GLP‐1RAs, biphasic insulin, or basal insulin combined with a DPP‐4 inhibitors is also advisable;^60^, ^113^, ^116^, ^117^ (3) Attempt to switch from premixed insulin to basal insulin to simplify the regimen and decrease the risk of hypoglycemia.

### 10.10 Fixed combination therapy

As T2DM progresses, combination therapy is often required. Fixed‐dose combination (FDC) and fixed‐ratio combination (FRC) are combination of two or more drugs in a fixed dose or ratio, which are important forms of combined medication. FDC and FRC have many advantages, such as suitability for a variety of pathophysiological mechanisms, simplified regimens, and diminished medication burdens on patients.^118^ These regimens usually improve compliance and satisfaction among older patients. Because the clinical advantages and limitations of fixed combination therapies depend on the components, their clinical use should be based on considerations of the overall condition of older patients. Currently approved fixed combination therapies in China include metformin‐based FDC, as well as FRC of basal insulin and GLP‐1RAs.

Several studies have examined fixed combination therapies in the older population. The GIFT study has indicated that switching from a co‐administered dual therapy with metformin and DPP‐4 inhibitors to an FDC regimen of the two improves glycemic control and achieves more significant blood glucose improvement in older patients, probably because of increased compliance.^119^ The LixiLan series studies and the DUAL series studies have verified the efficacy and safety of Insulin Glargine–Lixisenatide FRC and Insulin Degludec–Liraglutide FRC, respectively, in patients with T2DM. These FRC regimens have been confirmed to improve glucose levels in patients whose glycemic control cannot be well regulated with oral glycemic drugs, basal insulin, or GLP‐1RAs.^120^, ^121^, ^122^, ^123^, ^124^, ^125^ Post hoc analysis of the LixiLan‐L/O study and multiple DUAL series studies have indicated that, compared with therapies with a single component, Insulin Glargine–Lixisenatide FRC and Insulin Degludec–Liraglutide FRC are effective, safe, and tolerable in older patients.^116^, ^117^, ^126^

### 10.11 Recent advancements in glycemic drugs

In recent years, two newly approved classes of glycemic drugs with novel mechanisms of action have been introduced in China for the treatment of T2DM: peroxisome proliferator‐activated receptor (PPAR) pan‐agonists and glucokinase activators. One representative PPAR pan‐agonist is chiglitazar sodium, a next‐generation non‐TZD insulin sensitizer. This drug simultaneously activates the PPAR‐α, γ, and δ subtype receptors, thereby enhancing insulin sensitivity and consequently lowering blood glucose levels.^127^ A representative glucokinase activator is dorzagliatin, which regulates glucokinase activity in a glucose‐dependent manner, thus improving blood glucose regulation stability and exhibiting glycemic effects.^128^ Currently, clinical application of these two classes of drugs specifically in older patients with T2DM remains limited; therefore, more data is needed to demonstrate their effectiveness and safety in older patients with T2DM.

### 10.12 Treatment pathways

**FIGURE 1:**
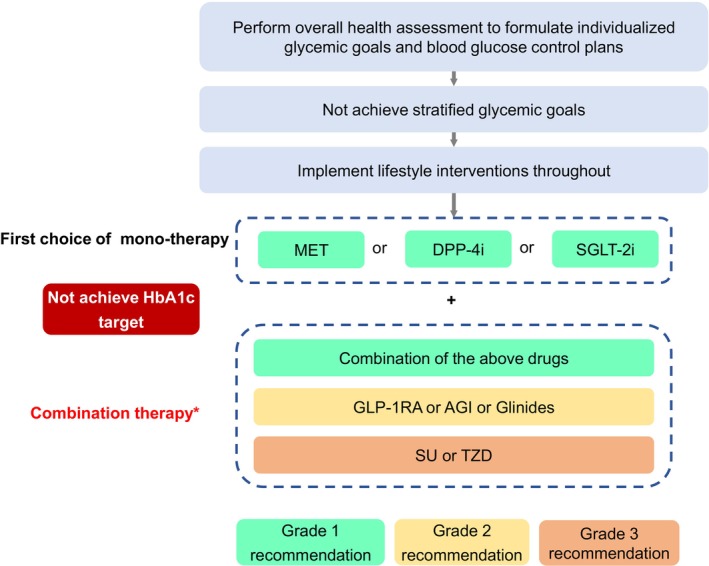
Pathway of non‐insulin therapies for older T2DM patients without established/high risk of ASCVD, HF, or CKD. AGI, α‐glycosidase inhibitor; ASCVD, atherosclerotic cardiovascular disease; CKD, chronic kidney disease; DPP‑4i, dipeptidyl peptidase IV inhibitor; GLP‐1RA, glucagon‐like peptide‐1 receptor agonist; HbA_1c_, glycated hemoglobin; HF, heart failure; MET, metformin; SGLT‐2i, sodium‐glucose cotransporter 2 inhibitor; SU, sulfonylureas; T2DM, Type 2 diabetes mellitus; TZD, thiazolidinediones. *As DPP‐4i and GLP‐1RA are both incretin drugs, glinides and SU are both insulin secretagogues; therefore, combination therapies of DPP‐4i and GLP‐1RA or glinides and SU should be avoided. The treatment pathway is intended for elderly patients with good (Group 1) and intermediate (Group 2) health status.

**FIGURE 2:**
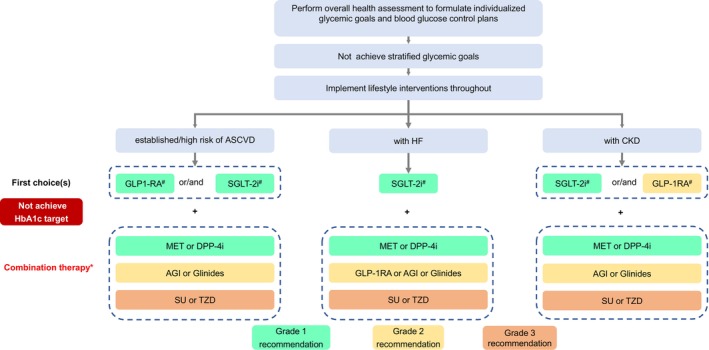
Pathway of non‐insulin therapies for older T2DM patients with established/high risk of ASCVD, HF, or CKD. AGI, α‐glycosidase inhibitor; ASCVD, atherosclerotic cardiovascular disease; CKD, chronic kidney disease; DPP‑4i, dipeptidyl peptidase IV inhibitor; GLP‐1RA, glucagon‐like peptide‐1 receptor agonist; HbA_1c_, glycated hemoglobin; HF, heart failure; MET, metformin; SGLT‐2i, sodium‐glucose cotransporter 2 inhibitor; SU, sulfonylureas; T2DM, Type 2 diabetes mellitus; TZD, thiazolidinediones. *As DPP‐4is and GLP‐1RAs are both incretin drugs, glinides and SU are both insulin secretagogues; therefore, combination therapies of DPP‐4i and GLP‐1RA or glinides and SU should be avoided. #Drugs with evidence for clinical benefits should be preferred. The pathway is intended for elderly patients with good (Group 1) and intermediate (Group 2) health status.

**FIGURE 3:**
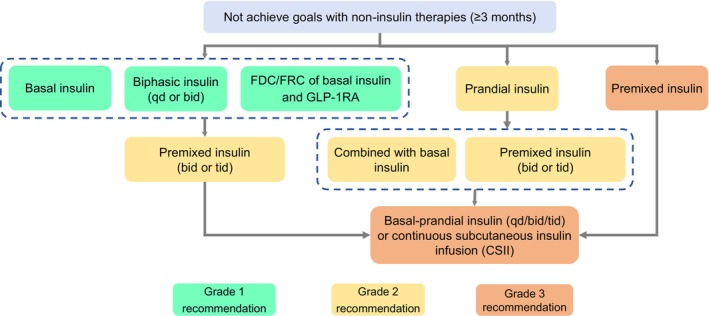
Pathway of insulin therapies for older T2DM patients. GLP‐1RA, glucagon‐like peptide‐1 receptor agonist; T2DM, Type 2 diabetes mellitus. Insulin therapy can be combined with noninsulin therapies. However, it is not advised to combine insulin with sulfonylureas or glinides. In terms of insulin, both human insulin and insulin analogues are included, and insulin analogues should be preferred. Only premixed insulin analogues can be administrated three times a day, but premixed human insulin or biphasic insulin cannot. The treatment pathway is intended for elderly patients with good (Group 1) and intermediate (Group 2) health status.

**FIGURE 4:**
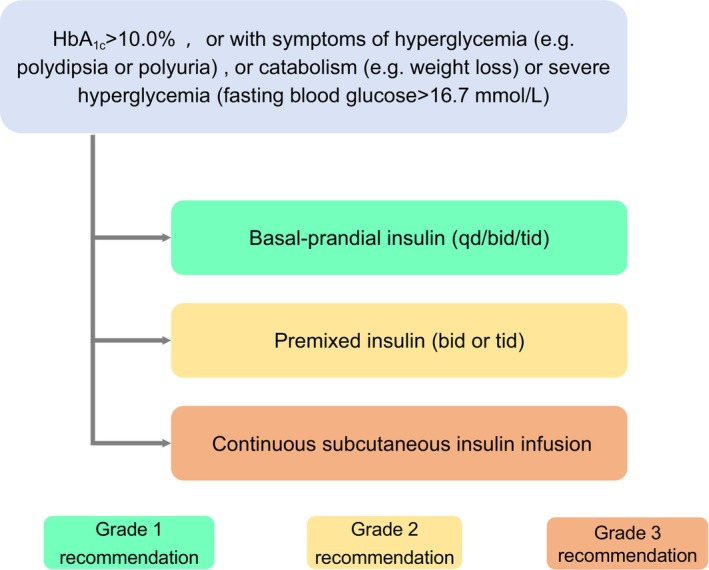
Pathway of short‐term insulin therapies for older T2DM patients. HbA_1c_, glycated hemoglobin; T2DM, Type 2 diabetes mellitus. In terms of insulin, both human insulin and insulin analogues are included, and insulin analogues should be preferred. Premixed insulin analogues can be administered three times a day, whereas premixed human insulin cannot. Non‐insulin therapy may be discontinued during the short‐term insulin therapy. Once the hyperglycemic state is relieved, the treatment strategy should be re‐evaluated and optimized.

Therapeutic drug choices should be based on the health status of older patients. Those with good (Group 1) or intermediate (Group 2) overall health assessment results require individualized glycemic therapies according to the presence of established/high risk of ASCVD, HF, or CKD. The treatment pathways may include non‐insulin therapies for (1) older patients with T2DM without established/high risk of ASCVD, HF, or CKD (Figure 1), or (2) older patients with established/high risk of ASCVD, HF, or CKD (Figure 2), as well as (3) insulin therapies for older patients with T2DM (Figure 3). The short‐term insulin treatment pathway for older patients with T2DM is shown in Figure 4. If the glycemic goal is not achieved after single‐drug therapy for more than 3 months, combination therapy with drugs with different mechanisms should be considered. However, caution is advised to avoid combining drugs that may increase the risk of hypoglycemia and other adverse events. For older patients who do not reach glycemic goals with standardized non‐insulin treatments, insulin therapy should be initiated promptly. The insulin treatment plan should include comprehensive patient education on preventing and managing hypoglycemia and proper insulin injection techniques to minimize the occurrence of hypoglycemia and other adverse events associated with insulin injection.

For patients with poor health status (Group 3), including those in an end‐of‐life stage, following the above pathways for treatment selection is not recommended. Instead, the approach should be based on aspects including these patients' vital organ function, drug treatment response, and hypoglycemia risk, to establish relatively loose glycemic goals. The fundamental principles include avoiding both hypoglycemia and severe hyperglycemia. Respecting the wishes of patients and their families, and selecting an appropriate glycemic therapy accordingly is essential. The use of oral drugs and/or long‐acting/ultra‐long‐acting basal insulins with a lower risk of hypoglycemia, such as Insulin Glargine U100, Insulin Degludec, and Insulin Glargine U300, is considered safer than multiple daily injections of rapid‐acting insulin or premixed insulin. This approach also facilitates easy dosage adjustment. The use of drugs with high risk of hypoglycemia and substantial weight loss is not recommended.

## 11 CHAPTER 9: ASCVD AND RISK FACTOR MANAGEMENT IN OLDER PATIENTS WITH DIABETES

**Key points**

1. In older patients with diabetes mellitus with established/high risk of ASCVD, glucose‐lowering medications should preferentially include GLP‐1RAs with cardiovascular protective effects or SGLT‐2 inhibitors. (A)
2. The control goal of systolic blood pressure (SBP) in older patients with diabetes is below 130 mmHg; however, vigilance is necessary regarding the risk of hypotension. (B)
3. Angiotensin‐converting enzyme inhibitors (ACEIs) or angiotensin II receptor blockers (ARBs) are preferred for the treatment of hypertension and should not be co‐administered; calcium channel blockers, diuretics, and β‐blockers are drug candidates for potential co‐administration with ACEIs or ARBs. (A)
4. Statin medications can decrease the risk of cardiovascular events in older patients with diabetes mellitus. Adding statin therapy is recommended for older patients with diabetes mellitus. However, high‐quality evidence regarding the optimal lipid control targets for older patients with diabetes mellitus remains lacking. Additionally, attention should be paid to the potential adverse reactions that may occur with statin therapy. (B)
5. For older patients with diabetes, routine use of aspirin is not recommended for primary prevention, but low dose (75–150 mg day^−1^) aspirin is recommended for secondary prevention. (A)
6. Older patients with diabetes are encouraged to actively cease smoking to decrease the risk of ASCVD. (A)
7. Body weight management in older patients with diabetes should consider both the body mass index and body composition. (B)

ASCVD includes coronary artery disease, cerebrovascular disease, and peripheral vascular disease caused by atherosclerosis and is a major cause of disability and death among people with T2DM.^129^ The risk of cardiovascular disease in patients with T2DM is more than twice that in people without diabetes.^130^ In clinical practice, actively screening and treating risk factors for cardiovascular disease is important. For older patients with diabetes mellitus who have concurrent established/high risk of ASCVD, priority should be given to glucose‐lowering medications with cardiovascular protective effects, including GLP‐1RAs or SGLT‐2 inhibitors with evidence‐based medicine support. Age itself is a risk factor for ASCVD, and ASCVD is also an important cause of disability and death in older people without diabetes. Furthermore, smoking, obesity and overweight, hypertension, and dyslipidemia are all important risk factors that contribute to the occurrence of ASCVD in older patients with diabetes. Most older patients with diabetes have multiple co‐existing cardiovascular risk factors and/or cardiovascular disease and kidney disease. However, older people are a heterogeneous group, and clinical research tends to exclude older patients with diabetes with advanced age and poor physical status; consequently, relevant data on older patients with diabetes are limited, and no consensus has been reached regarding the risk factors for ASCVD.

### 11.1 Screening and evaluation

Multiple macrovascular complications may progress for years before the diagnosis of diabetes, thus making the management of ASCVD challenging. Consequently, active screening for ASCVD and related risk factors is critical. Patients are recommended to undergo blood pressure monitoring at each visit, and to undergo systemic evaluation of ASCVD risk factors (including overweight and obesity, hypertension, dyslipidemia, smoking, and family history of early onset coronary heart disease, CKD, and proteinuria) at least annually; for older patients with diabetes with the above ASCVD risk factors, ultrasonic evaluation of the carotid arteries and lower extremity arteries should be actively performed to assess any peripheral vascular disease, so that the risk factors can be identified and intervention can occur as early as possible.

### 11.2 Cardiovascular risk factor management

1. Hypertension: Older patients with diabetes have elevated risk of hypertension, which is an independent risk factor for cardiovascular disease. After other risk factors are controlled, every 10 mmHg (1 mmHg = 0.133 kPa) increase in SBP is associated with a 30% higher risk of ischemic heart disease and ischemic stroke,^131^ whereas anti‐hypertensive therapy can decrease the occurrence and death risks of cardiovascular events in patients with diabetes.^132^ In older patients, the benefits of anti‐hypertensive therapy have also been extensively demonstrated in clinical studies.^133^
  1. Control goal: Currently, research specifically examining blood pressure control targets for older patients with diabetes mellitus is lacking. The blood pressure target for older patients with diabetes mellitus should be individualized, considering the benefit‐to‐risk ratio. In the ACCORD BP study, an intensive blood pressure control (SBP target <120 mmHg), compared with standard blood pressure control (SBP target <140 mmHg), did not decrease the primary composite endpoint (nonfatal myocardial infarction, nonfatal stroke, and cardiovascular death), but increased the risk of adverse events such as hypotension and hyperkalemia.^134^ Results from the STEP study conducted in China and published in 2021 have indicated that, compared with standard blood pressure control (SBP target 130–150 mmHg), intensive blood pressure control (SBP target 110–130 mmHg) decreases the risk of MACE by 26% in older patients without increasing the risk of adverse events such as dizziness or fractures, except for an increased risk of hypotension. In that study, 18.9% and 19.4% of patients in the intensive and standard blood pressure control groups, respectively, were older patients with diabetes mellitus (60–80 years of age).^135^ The SBP target for older patients with diabetes mellitus is recommended to be set below 130 mmHg to decrease the risk of cardiovascular disease.^136^ However, blood pressure should be closely monitored to prevent conditions such as orthostatic hypotension, postprandial hypotension, and excessively low diastolic blood pressure. A SBP < 120 mmHg is not recommended as a control goal of older patients with diabetes.^137^ For patients who are 80 years or older or have a short life expectancy or poor health status (Group 3), the SBP control goal can be appropriately extended to below 150 mmHg.^2^
  2. Drug choice: ACEIs significantly decrease major cardiovascular adverse events, cardiovascular deaths, and all‐cause mortality among patients with diabetes^138^; and in older patients with diabetes, ACEIs also decrease cardiovascular deaths.^139^ ARBs have similar effects in patients with diabetes^140^; and in older people, ARBs significantly decrease stroke.^141^ ACEIs or ARBs are recommended as the first‐line medications for controlling blood pressure in older patients with diabetes^2^ but should not be co‐administered^140^; otherwise, hyperpotassemia and acute kidney injury may occur.^141^ Blood potassium and creatinine levels should be closely monitored during administration. If the blood pressure is inadequately controlled by ACEIs or ARBs alone, calcium channel blockers (CCBs), thiazide diuretics, or β‐blockers may be added for lowering blood pressure.^2^
2. Dyslipidemia: Older patients with diabetes often have dyslipidemia, thus leading to an increased risk of ASCVD. Limited medical evidence supporting lipid lowering therapy in older patients with diabetes is available. On the basis of existing evidence, controlling the LDL‐C of older patients with diabetes below 2.6 mmol L^−1^,^142^, ^143^ and controlling that of older patients with diabetes and concurrent ASCVD below 1.8 mmol L^−1^,^142^, ^143^ are recommended; however, for patients of 80 years or older, or those with a short life expectancy or poor health status (Group 3), the LDL‐C goals should be appropriately relaxed. Statins can decrease the risk of cardiovascular events^144^, ^145^ and all‐cause mortality in older patients.^145^, ^146^ Both the Heart Protection Study DM Subgroup (HPS‐DIM) and Collaborative Atorvastatin Diabetes Study (CARDS) have suggested that statins decrease the risk of cardiovascular events in older patients with diabetes.^147^, ^148^ However, evidence in older patients (80 years or older) with diabetes remains lacking. Studies have shown that ezetimibe plus simvastatin decreases ischemic stroke risk in patients with acute coronary syndrome by 24%,^149^ and this benefit is enhanced in patients with concurrent diabetes or other risk factors.^150^ Older patients with diabetes are recommended to use statins to decrease cardiovascular events and all‐cause mortality. If the LDL‐C goal can not be achieved with statins alone, ezetimibe or PCSK9 inhibitors may be added with caution.^2^, ^142^, ^143^ For older patients with diabetes whose triglycerides exceed 5.65 mmol L^−1^, fenofibrate may be used to decrease pancreatitis risk, but caution is necessary regarding the increased risk of adverse events when fenofibrate is concomitantly used with statins. Given that older patients with diabetes often have multiple concurrent diseases requiring polymedication, close attention should be paid to monitoring the safety of statins and their interactions with other drugs, and to monitoring changes in liver function and creatine kinase levels.
3. Antiplatelet therapy: The benefits and risks of aspirin antiplatelet therapy should be weighed according to the bleeding risk, risk of underlying cardiovascular disease, compliance with aspirin therapy, and age. Although primary prevention with aspirin decreases cardiovascular events in patients with diabetes, it increases the risk of bleeding events, and older age is associated with higher bleeding risk. According to Aspirin in Reducing Events in the Elderly (ASPREE), in the population of 70 years or older with the risk of cardiovascular disease, aspirin increases the risk of bleeding instead of decreasing the incidence of cardiovascular disease.^151^ Recent meta‐analyses have shown similar results.^152^ At present, no sufficient evidence indicates that the benefits of aspirin outweigh the risks for primary prevention in older people with diabetes; therefore, routine use of aspirin in these patients for primary prevention of cardiovascular events is not recommended. Older patients with diabetes and concurrent ASCVD are recommended to use low‐dose aspirin (75–150 mg day^−1^) for secondary prevention.^2^ However, patients of 80 years or older, or those with a short life expectancy or poor health status (Group 3), should be managed individually. Because the most common adverse event of aspirin is gastrointestinal hemorrhage, bleeding risk should be fully evaluated before use. The risk of bleeding is associated with factors including high‐dose of aspirin, long‐term administration, severe hepatic insufficiency, renal insufficiency, peptic ulcers, hemorrhagic diseases, diminished platelets, use of non‐steroidal anti‐inflammatory drugs, and inadequately controlled blood pressure. After aspirin use, patients and their families should be fully educated to identify possible bleeding risks in a timely manner. In addition, concomitant use of proton pump inhibitors (PPIs) may help decrease the risk of gastrointestinal hemorrhage.
4. Cessation of smoking: Smoking increases the risk and mortality of coronary heart disease and stroke, which shows a dose–response relationship^153^, ^154^; moreover, passive smoking also increases the risk of cardiovascular disease.^155^, ^156^ In older people, smoking remains an important independent risk factor for cardiovascular disease, and smoking cessation can help decrease the risk of cardiovascular disease. Because people of all ages can benefit from cessation of smoking, older patients with diabetes should be actively encouraged to cease smoking.^157^
5. Body weight management: Obesity is associated with various cardiovascular diseases and can directly or indirectly increase cardiovascular disease incidence and mortality. Weight loss can improve blood glucose control and reduce other cardiovascular risk factors. However, weight loss interventions in older patients with diabetes mellitus should consider the their characteristics, such as body composition and risks of malnutrition, sarcopenia, and osteoporosis. As individuals age, they experience changes characterized by muscle loss and increased fat deposition. The body mass index (BMI) has limitations in reflecting obesity in older adults. When assessing obesity in older individuals, considering the BMI, the waist circumference, waist‐to‐hip ratio, and body muscle mass is recommended. A comprehensive evaluation of body weight, body composition, and muscle function should be conducted to develop weight management strategies. Dietary interventions for weight loss should avoid malnutrition and appropriately increase protein intake. Weight loss through diet alone without resistance exercise can lead to loss of lean tissue (muscle and bone), thus exacerbating age‐associated sarcopenia and loss of bone mass.^158^ Encouraging older patients with diabetes mellitus to engage in resistance training is important to increase body muscle mass. For older patients with both obesity and diabetes, choosing medications that can achieve both weight loss and glucose lowering is recommended. GLP‐1 RAs (such as liraglutide, dulaglutide, and semaglutide) with evidence of strong weight loss intensity and cardiovascular protection should be preferred. If those medications are not tolerated, SGLT‐2 inhibitors with moderate intensity of weight loss can be considered as an alternative.

## 12 CHAPTER 10: SCREENING AND TREATMENT OF CHRONIC COMPLICATIONS OF DIABETES IN OLDER PEOPLE

### 12.1 Diabetic kidney disease

**Key points**

1. Older patients with diabetes should undergo renal function assessment at the time of diabetes are diagnosed and thereafter on a yearly basis. (A)
2. The degree of nephropathy should be screened and evaluated with a combination of two methods: urine albumin/creatinine ratio and eGFR. (A)
3. SGLT2 inhibitors are preferred for older patients with diabetes and CKD. In cases of intolerance or contraindications, GLP‐1RAs with evidence of renal protection should be considered. (B)
4. ACEIs or ARBs treatment should be used for anti‐hypertensive therapy. (B)
5. The non‐steroidal mineralocorticoid receptor antagonist finerenone is recommended to delay the progression of kidney disease in older patients with diabetes and concomitant CKD and proteinuria. (A)
6. Chronic kidney disease in older patients with diabetes is usually caused by a variety of factors, and integrated management based on glucose‐lowering therapy should be undertaken. (A)

Diabetic kidney disease (DKD) is the major cause of CKD in China.^159^ Some people with T1DM develop DKD after a disease course of 5 to 10 years, whereas patients with T2DM might have nephropathy at the time of diagnosis. If not controlled, DKD can eventually develop into end‐stage renal disease, thus severely affecting quality of life and increasing the medical burden. Furthermore, the risk of cardiovascular disease is also significantly elevated in people with DKD.

#### 12.1.1 Screening

At the time of diagnosis, older patients with diabetes should undergo screening and assessment of the urine albumin/creatinine ratio (UACR) and blood creatinine (used to calculate eGFR) to promote identification of early kidney damage. Patients should be followed up at different frequencies according to their renal function status (Table 5).^160^, ^161^

**TABLE 5:** Risk of CKD progression and visit frequency, classified by eGFR and UACR.

CKD categories	Degree of kidney damage	eGFR [mL min^−1^ (1.73 m^2^)^−1^]	Albuminuria categories		
			A1 (UACR <30 mg g^−1^)	A2 (UACR 30–300 mg g^−1^)	A3 (UACR >300 mg g^−1^)
Stage 1 (G1)	eGFR normal or high	≥90	1 (if combined CKD)	1	2
Stage 2 (G2)	eGFR mildly decreased	60–89	1 (if combined CKD)	1	2
Stage 3a (G3a)	eGFR mildly to moderately decreased	45–59	1	2	3
Stage 3b (G3b)	eGFR moderately to severely decreased	30–44	2	3	3
Stage 4 (G4)	eGFR severely decreased	15–29	3	3	4
Stage 5 (G5)	Kidney failure	<15	4	4	4

*Note*: The background colors in the table indicate the risk of CKD progression, with green representing low risk, yellow representing moderate risk, orange representing high risk, and red representing very high risk. The numbers in the table suggest the recommended frequency of annual reexamination.

Abbreviations: eGFR, estimated glomerular filtration rate; CKD, chronic kidney disease; UACR, ratio of urinary albumin to creatinine.

Clinical diagnosis should be made according to increased UACR and/or decreased eGFR, and by ruling out CKD caused by other factors. Routine kidney aspiration biopsy is not recommended. Patients with DKD often have a long disease course and frequently have concurrent diabetic retinopathy (DR), manifesting primarily as proteinuria without gross hematuria, with a gradual decrease in eGFR. Notably, older people with diabetes who have diabetic renal damage usually have concurrent hypertension, hyperlipidemia, hyperuricemia, drug‐induced renal damage, and other factors; diabetes‐induced renal injury accounts for one‐third of renal injury in this patient group.^162^

UACR testing with random urine is the simplest screening method, and UACR >30 mg/g is considered elevated; however, strenuous exercise, infection, fever, congestive HF, and significant increases in blood glucose or blood pressure may also cause the UACR to increase. Some patients may develop renal impairment when the UACR remains within the normal range. eGFR is an important tool for evaluating renal function. In general, calculation of eGFR with the CKD Epidemiology Collaboration equation is recommended.^163^ Older people may have false normal eGFR findings because of low body weight and inadequate protein intake; consequently, the significance of renal function assessment based on the eGFR alone in older people is limited, and the definition of eGFR in older people remains controversial.^164^

#### 12.1.2 Treatment

Integrated treatment based on glucose‐lowering therapy should be emphasized for older patients with diabetes with concurrent CKD.

1. For patients not receiving dialysis, the recommended daily intake of high‐quality protein is 0.8 g kg^−1^. Limiting sodium salt intake is recommended because sodium chloride <5 g day^−1^ or sodium <2 g day^−1^ can help lower blood pressure and decrease the risk of cardiovascular disease.^165^
2. For glucose‐lowering therapy, SGLT2 inhibitors are preferred for older patients with diabetes and CKD. In cases of intolerance or contraindications, GLP‐1RAs with evidence of renal protection should be considered, followed by drugs that are essentially not excreted via the kidney, such as linagliptin, repaglinide, and gliquidone. During the use of glucose‐lowering medications, attention should be paid to whether dose adjustment is required, according to the eGFR, and the onset of hypoglycemia should be monitored.
3. For anti‐hypertensive therapy, ACEI or ARB can be chosen. If necessary, these drugs can be co‐administered with other types of anti‐hypertensive drugs. During the administration, attention should be paid to monitoring blood pressure, renal function, and blood potassium. The achievement of blood pressure control goals is crucial for delaying the progression of kidney disease. The FIDELIO‐DKD study has demonstrated a significant decrease in the risk of renal composite endpoint events in patients with T2DM and CKD treated with finerenone.^166^ The results from the Chinese subgroup data are consistent with those in the overall population.^167^ The FIDELITY study, a predefined pooled analysis of two large international multicenter phase III trials (FIDELIO‐DKD and FIGARO‐DKD^168^), has revealed that finerenone significantly decreases the long‐term risk of renal and cardiac composite events in older individuals with T2DM and CKD, in agreement with findings in the overall population.^169^ For older patients with diabetes with CKD and proteinuria (eGFR ≥25 mL min^−1^ (1.73 m^2^)^−1^), adding finerenone to the maximum tolerated dose of ACEI or ARB is recommended to decrease proteinuria and delay CKD progression. Monitoring of serum potassium and renal function is essential. Additionally, a few cases of severe anemia have been reported.
4. Smoking should be ceased, and blood lipid and uric acid levels should be controlled.
5. Collaborative multidisciplinary management with a nephrologist is recommended for older patients with diabetes with combined CKD.
6. Drugs should be used with caution, unnecessary use of Chinese and Western medicines should be avoided, and patients should particularly be warned not to use so‐called kidney‐protecting drugs.

### 12.2 Diabetic eye diseases

**Key points**

1. Older patients with diabetes should be screened for diabetic fundus lesions at the time of diagnosis and once yearly thereafter; if fundus lesions are found, the frequency of reexamination should be increased. (A)
2. Beyond the fundus, attention should be paid to abnormalities in visual acuity, intraocular pressure, and the ocular surface in older patients with diabetes. (C)

Diabetes is associated with a variety of eye diseases. Diabetic eye diseases may cause visual acuity decrease or even blindness and may make older people with diabetes unable to participate in social activities; compromise their capability of fingertip pricking for glucose measurement and insulin injection; and increase their risk of accidents. Older patients with diabetes should undergo fundus examination at the time of diagnosis, should visit ophthalmologists for thorough checkups as needed, and should be screened at least once per year thereafter. Annual screenings should include visual acuity, intraocular pressure, ocular surface, and fundus examination, with a focus on diabetic retinopathy (DR) and macular edema. Additionally, assessment for cataracts, glaucoma, and dry eye syndrome should be performed.

#### 12.2.1 Diabetic fundus diseases

1. DR: DR, a common microvascular complication of diabetes, causes severe retinal damage and significantly affects quality of life. According to the disease stage, DR can be divided into non‐proliferative DR and proliferative DR. The manifestations of non‐proliferative DR include microaneurysm formation and intra‐retinal hemorrhage, wherein microvascular injury can increase vascular permeability (retinal edema and effusion); proliferative DR manifests as neovascularization in the optic disc, retina, iris, and chamber angle, thus eventually resulting in tractional retinal detachment and neovascular glaucoma. Routine DR screening is recommended for older patients with diabetes at the time of diagnosis. Subsequent annual follow‐ups are advised for those with no DR or mild non‐proliferative DR, or once every 6 months in cases of moderate non‐proliferative DR, or once every 3 months in cases of severe non‐proliferative DR and proliferative DR.^170^ Long disease courses and suboptimal glycemic control are the risk factors for the occurrence and progression of DR; in addition, albuminuria, hypertension, and hyperlipidemia are risk factors for DR. Therefore, improvements in blood glucose, blood pressure, and blood lipids may aid in decreasing the occurrence of DR. Meta‐analyses have indicated a significant association between GLP‐1RAs, as compared with a placebo, and the risk of early DR. However, in comparison to insulin, GLP‐1RAs have shown protective effects against advanced DR.^171^ A meta‐analysis of CVOT data on GLP‐1RAs has not found a direct association between GLP‐1RAs and DR, but has found a correlation with decreased HbA_1c_.^172^ Monitoring retinal status, particularly for patients with proliferative DR and/or severe DR, is essential during strict blood glucose management with GLP‐1RAs or other hypoglycemic agents. The relationship between GLP‐1RAs and DR may be influenced by factors such as the type of agonist, patient sex, age, and disease duration.^171^ Data specific to the older diabetic population regarding the association between GLP‐1RAs and DR are currently lacking. Pan‐retinal laser photocoagulation is the major treatment method for proliferative DR, and intravitreous anti‐vascular endothelial growth factor (VEGF) is another effective treatment.
2. Diabetic macular edema (DME): DME is a disease of fluid accumulation in the central fovea of the macula, resulting from blood–retina barrier failure, and leading to extensive capillary leakage and subsequently diffuse edema. DME is common in older people with diabetes and may be accompanied by DR or may occur alone; this condition is a major contributor to visual impairment in people with diabetes. Optical coherence tomography is the gold standard for diagnosing DME and can be used for screening, classification, monitoring, and assessing treatment efficacy. Fluorescein angiography provides information on vascular leakage and barrier function. Older patients with diabetes are recommended to undergo screening for DME by optical coherence tomography at the time of diagnosis and thereafter once yearly, and if necessary, to undergo fluorescein angiography. For clinically significant macular edema (located in the central macula or threatening the central macula), follow‐up examinations should be conducted every 3 months. In the past, focal/grid laser photocoagulation was the standard treatment for DME. In recent years, VEGF therapy has gained recognition, and the 2017 guidelines from the European Society of Retina Specialists recommend VEGF therapy as the first choice for treating macular edema involving the central fovea.^173^

#### 12.2.2 Other related eye diseases

T2DM is a risk factor for cataracts.^174^ Age is closely associated with cataracts, and cataracts are the main cause of blindness in older people.^175^ Glaucoma is the second leading cause of blindness in older people, and people with diabetes have a higher risk of glaucoma than those without diabetes.^176^ Examining visual acuity and intraocular pressure and screening for cataracts and glaucoma are particularly important in older patients with diabetes. Beyond eye disease that may decrease visual acuity, attention should also be paid to xerophthalmia in older people with diabetes. Xerophthalmia is the most prevalent ocular surface disease.^177^ Diabetes is significantly associated with the risk of xerophthalmia.^178^ A communitybased study in Shanghai has indicated a 17.5% prevalence of xerophthalmia in patients with T2DM with a mean age of 69 years.^179^ Xerophthalmia can lead to a variety of eye discomforts (e.g., dry eye, eye redness, sensation of sand or gravel in the eyes, burning sensation, foreign body sensation, excessive tearing, and photophobia) and visual impairment,^180^ and may contribute to decreased sleep quality, as well as the development of anxiety and depression.^181^ Older patients with diabetes should be asked about the presence of xerophthalmia symptoms, and further examinations and initiation of treatments should be performed if necessary.

### 12.3 Diabetic neuropathy

**Key points**

1. Older patients with diabetes should be screened for distal symmetric polyneuropathy (DSPN) at the time of diagnosis and yearly thereafter. (A)
2. Screening and treatment of diabetic autonomic neuropathy (DAN), particularly cardiac autonomic neuropathy (CAN), are important in older patients with diabetes. (B)
3. Diabetic painful neuropathy severely affects quality of life in older patients with diabetes and consequently should be considered seriously. (B)

Diabetic neuropathy, a common chronic complication of diabetes, is heterogeneous, affecting different central and peripheral nerves, and presenting as diverse clinical manifestations. DSPN is the most representative diabetic peripheral neuropathy. Beyond DSPN, DAN is also a common diabetic peripheral neuropathy. DAN can involve multiple systems, including the cardiovascular, gastrointestinal, urogenital, and sudomotor systems, thus resulting in an array of symptoms and signs, such as hypoglycemia unawareness, tachycardia at rest, postural hypotension, gastroparesis, constipation, diarrhea, erectile dysfunction, neurogenic bladder, and sweating disorders, among which CAN is the greatest concern. Independently of other cardiovascular risk factors, CAN is associated with mortality risk.^182^ In addition, gastrointestinal autonomic neuropathy manifests as symptoms including dysphagia, hiccups, gastroparesis, constipation, and diarrhea, which severely affect quality of life in older people with diabetes.

#### 12.3.1 DSPN

DSPN, the most common diabetic neuropathies type, accounting for approximately 75% of cases, is an important risk factor for diabetic foot ulcers and is also a major contributor to falls and fractures.^183^ Age is an independent risk factor for DSPN,^184^ but nationwide epidemiological survey data on older patients with diabetes with DSPN are currently lacking. In the Beijing region, data have indicated a prevalence of 42.6% for DSPN and of 26.2% for suspected DSPN among older individuals with T2DM.^185^ Clinically, DSPN manifests as numbness, pain, and paresthesia of the bilateral extremities. Newly diagnosed older patients with diabetes should undergo DSPN assessment, including a thorough medical history as well as assessment of small‐fiber function (temperature sensation and pricking pain sensation) and large‐fiber function (pressure sensation, ankle reflexes, and vibration sensation), with follow‐up once yearly thereafter. Moreover, these patients should undergo 10 g nylon monofilament testing every year to promote the early identification of the risk of foot ulcers and limb amputation. Currently, no effective treatment is available to reverse DSPN. After a diagnosis of DSPN is considered, treatment should be initiated as early as possible to delay DSPN progression. Glycemic control is the main means of preventing DSPN and delaying its progression. Comprehensive management addressing various risk factors such as blood pressure and lipid levels also contributes to delaying DSPN progression. For patients with DSPN, integrated management goals should be formulated to address the aforementioned risk factors. In addition, data suggest that treatments such as methylcobalamin for nutritional support to nerves, alpha‐lipoic acid for antioxidant stress, prostaglandin E1 for microcirculation improvement, and the aldose reductase inhibitor epalrestat have efficacy in treating older patients with DSPN.

#### 12.3.2 CAN

Nationwide epidemiological data on CAN in older individuals with diabetes are currently lacking. Data from the Beijing region indicate a high prevalence of CAN, affecting 55.7% of older patients with T2DM.^186^ The overall prevalence of CAN in T2DM is as high as 62.6%, and that in patients with T2DM ≥ 60 years of age is 67.5%, a value slightly higher than that in the general population. Multifactorial logistic regression analysis has identified age as an independent risk factor for CAN.^187^ For older patients with diabetes, attention to CAN is highly important. Older patients with diabetes who have microvascular and neurological complications and hypoglycemia unawareness should be assessed for CAN symptoms or signs. Furthermore, people with diabetes with greater glycemic variability are more likely to develop CAN.^188^ In early stages of CAN, people may be asymptomatic and may show decreased heart rate variability only during physical examination; as the disease progresses, these individuals may develop tachycardia at rest and postural hypotension (with common symptoms including dizziness and asthenia), or even syncope and painless myocardial infarction. Cardiovascular reflex tests, heart rate variability, blood pressure measurement during position changes, and 24‐h ambulatory blood pressure monitoring can aid in confirming the diagnosis. If CAN is suspected, other concurrent diseases or concomitant drugs that may affect the cardiac autonomic nervous function should be further excluded. Patients with CAN and their families should be educated to prevent falls and avoid the occurrence of hypoglycemia, particularly nocturnal hypoglycemia. Research has indicated that combining aerobic exercise with resistance training improves heart rate variability in middle‐aged and older female patients with T2DM, thus ameliorating CAN.^189^ Medications such as midodrine, targeting the sympathetic nervous system, can be considered for treating orthostatic hypotension in older patients with diabetes.^190^

#### 12.3.3 Diabetic painful neuropathy

Diabetic neuropathy may result in neuropathic pain through a variety of pathogenic mechanisms. Neuropathic pain typically manifests as burning pain, pricking pain, or shooting (electric shock) pain, accompanied by paresthesia, and may include multiple simultaneous symptoms that worsen in the nighttime. This pain disturbs daily life, jeopardizes quality of life, and may even cause disability and mental illness. Assessment and treatment of sleep and mood disorders are crucial. The most recent research from the American Neurological Association indicates that gabapentinoids, serotonin‐norepinephrine reuptake inhibitors, sodium channel blockers, and tricyclic antidepressants (TCAs) can be used to treat pain caused by diabetic peripheral neuropathy.^191^ Head‐to‐head trials have demonstrated therapeutic equivalence of gabapentinoids, serotonin‐norepinephrine reuptake inhibitors, and TCAs in treating DPN pain, with combination therapy being superior to monotherapy.^192^ Because of the substantial incidence of adverse reactions associated with TCA medications, attention should be paid when these drugs are used in older patients with diabetes.

### 12.4 Lower extremity arterial disease and diabetic foot

**Key points**

1. Older patients with diabetes have an elevated incidence of lower extremity atherosclerotic disease, and patients with lower extremity atherosclerotic disease have an elevated risk of cerebro‐cardiovascular events and diabetic foot. (B)
2. For diabetic foot, prevention is better than treatment. (A)
3. The prognosis of diabetic foot can be improved by early identification of risk factors, intensive patient and family education, and interdisciplinary cooperative management. (B)

Lower extremity arterial disease (LEAD) is a peripheral arterial disease manifested as stenosis or occlusion of the lower extremity arteries. Normally, LEAD in patients with diabetes refers to lower extremity atherosclerotic disease. According to the China DIA‐LEAD study, the overall prevalence of LEAD in patients with T2DM 50 years or older in China is 21.2%, and the prevalence increases with age and the course of diabetes.^193^ In patients with T2DM with LEAD, age is independently associated with lesion severity.^194^ The risks of death due to myocardial infarction, stroke, and coronary heart disease are all elevated in patients with LEAD.^195^ LEAD is also a major risk factor for diabetic foot. Vasodilators such as cilostazol, sarpogrelate, pentoxifylline, and prostaglandins can ameliorate ischemic symptoms in the lower limbs; however, for patients with LEAD who do not respond to medical treatment, experience severe intermittent claudication affecting the quality of life, or have skin ulcers or gangrene, revascularization may be considered appropriate to maintain their functional status and independent ADL.

Diabetic foot refers to foot infection, ulcer, or even deep tissue damage caused by distal neuropathy and vasculopathy of the lower extremities in people with diabetes. This condition is one of the most serious chronic complications of diabetes, and severe cases can lead to amputation and death. Diabetic foot is associated with a variety of factors, predominantly peripheral arteriopathy and peripheral neuropathy; such factors also include trauma, infection, excessively high foot pressure due to foot deformity, and limited joint motion. Foot ulcers in Chinese patients with diabetes are primarily neuropathic and ischemic ulcers, or in rare cases are simple neuropathic ulcers or ulcers caused by Charcot foot deformity.^196^ Because of poor vision, difficulty walking, and difficulty bending from the waist, older people with diabetes often have difficulty in self‐examination or self‐care of their feet, thus making early detection of foot problems difficult. Therefore, these individuals have elevated risk of diabetic foot, and make up most of the population with diabetic foot in China.^197^

The management of diabetic foot emphasizes that prevention is better than treatment. In the prevention of diabetic foot, attention should be paid to examining and eliminating risk factors for diabetic foot, educating patients and their families, and actively seeking interdisciplinary collaboration. The assessments and examinations include foot ulcer history taking and determining whether shoes are appropriate; whether protective sensation is lost; whether any vascular dysfunction is present (pulsation of dorsal pedal artery and posterior tibial artery); and whether foot deformity, callus, or pre‐ulcerative lesion is present. Early identification and elimination of the above risk factors is the major measure to prevent diabetic foot ulcer. Education regarding diabetic foot can decrease the incidence^198^ and recurrence rates of diabetic foot ulcers. The education should include instructing patients to periodically examine their feet, particularly the interdigital spaces; periodically wash the feet and dry the interdigital spaces; avoid wearing tight socks or shoes; and check the shoes for any foreign bodies inside before donning.

Older patients with diabetes should pay greater attention to foot care by^199^: (1) checking the feet including the interdigital spaces every day, and if necessary, seeking help from the family or nursing personnel; (2) avoiding scalding and burning; (3) applying emollient oil or cream to dry skin, but not in the interdigital spaces; (4) directly clipping the nails crosswise, and trimming the edges and corners with a nail file; (5) avoiding use of any chemical agent or plaster to remove corns and calluses; (6) checking the shoes for any foreign bodies inside before donning; (7) avoiding walking barefoot; (8) undergoing periodic foot examinations by HCPs; (9) and seeking medical attention immediately if a foot skin blister, cut, scrape, or sore is found.

The foot condition of older patients with diabetes must be actively assessed. For patients with diabetic foot, differentiating the etiology of ulcers (ischemic, neuropathic, or neuro‐ischemic), the nature of gangrene (wet gangrene or dry gangrene), and performing diabetic foot classification (Wagner classification, Texas classification, etc.) are important. In China, the neuro‐ischemic type of diabetic foot is more common, and treatment should focus on treating neuropathy, exercising, and reconstructing lower limb blood flow. Limb decompression, local debridement, and promotion of ulcer healing are essential. In cases of diabetic foot with infection, active anti‐infective measures should be taken. After the occurrence of conditions such as a sharp change in skin color, aggravation of local pain with manifestations of inflammation such as redness and swelling, new ulcers, deterioration of an original superficial ulcer involving soft tissue and/or bone tissue, disseminated cellulitis, systemic infection signs, and osteomyelitis, prompt internal medicine treatment should be combined with assistance from vascular surgery, orthopedics, wound surgery, and other relevant specialties, and surgical treatment should be performed if necessary. Timely referral or multidisciplinary diagnosis and management can help improve the healing rate of foot ulcer and decrease the amputation rate.^200^

## 13 CHAPTER 11: ACUTE COMPLICATIONS OF DIABETES IN THE ELDERLY

**Key points**

1. Hypoglycemia is an acute complication which older patients with diabetes should be especially vigilant of. To avoid hypoglycemia, reasonably individualized glycemic targets should be established, and glucose‐lowering medications with low risk of hypoglycemia should be selected. (A)
2. The hypoglycemia risk of elderly patients with T1DM may be reduced by wearing a continuous glucose monitoring system. (A)
3. The hyperglycemic crisis in older patients with diabetes often exists as a hyperglycemic hyperosmolar state, with the case fatality much higher than that of diabetic ketoacidosis. Fluid replacement is an important treatment approach, insulin should be used to lower blood glucose, and attention should be paid to potassium supplements. (B)
4. Lactic acidosis has a low morbidity but a high mortality and thereby should be taken seriously. (B)

Hypoglycemia, hyperglycemic hyperosmolar state (HHS), and diabetic ketoacidosis (DKA) are severe acute complications of diabetes, requiring rapid identification, timely diagnosis, and active treatment. Hypoglycemia is an adverse reaction in the course of glucose‐lowering therapy that can result in short‐term and long‐term adverse clinical outcomes and increase the mortality. Compared to non‐elderly patients, older patients with diabetes are more likely to develop hypoglycemia and are at higher risk of mortality associated with hypoglycemia, requiring much attention from clinicians. DKA and HHS are characterized by insulin deficiency and severe hyperglycemia. Clinically, these two conditions only differ in the degree of dehydration and severity of metabolic acidosis. The prognosis and outcome of DKA or HHS patients depends on the age, severity of dehydration, comorbidity, and whether the disease is treated in a timely and standardized manner. The prognosis and outcome of older patients with diabetes who have DKA and HHS are usually worse than those younger patients with diabetes.

### 13.1 Hypoglycemia

In older patients with diabetes, hypoglycemia is one of the common acute complications, resulting in arrhythmia, myocardial infarction, falls, and even coma, death, and other adverse events. Repeated episodes of severe hypoglycemia can cause cognitive decline and even dementia in these patients.^201^

1. Define and standard: Currently, both domestic and international guidelines define and classify hypoglycemia as follows: For diabetes patients undergoing drug treatment, blood glucose levels below 3.9 mmol L^−1^ are considered hypoglycemic. (1) Grade 1 hypoglycemia: Blood glucose <3.9 mmol L^−1^ and ≥3.0 mmol L^−1^. (2) Grade 2 hypoglycemia: Blood glucose <3.0 mmol L^−1^. (3) Grade 3 hypoglycemia: Severe events requiring assistance from others, accompanied by changes in consciousness and/or body, but without specific blood glucose limits.^202^ Due to the strong heterogeneity of the elderly population and the lack of hypoglycemia studies specifically in this group, coupled with the absence of unified diagnostic criteria for hypoglycemia in different studies, there is a lack of large‐scale epidemiological data on the incidence of hypoglycemia in elderly diabetes patients in China.
2. Risk factor: Age is one of the risk factors for hypoglycemia^203^; the risk of hypoglycemia is higher in older patients with diabetes than in non‐elderly ones.^204^ In addition to the age actor, compromised ability of glucose regulation, multiple concurrent diseases (e.g., CKD, cardiovascular disease, hepatic insufficiency, etc.), polypharmacy, and concurrent autonomic neuropathy are all risk factors for hypoglycemia in older patients with diabetes.^205^ Cognitive decline is also an important contributor to the increased risk of severe hypoglycemia in older patients with diabetes.^206^ Unhealthy living habits such as fasting alcohol consumption, excessive carbohydrate restriction, irregular eating, and no extra meals before heavy exercise are common triggers for hypoglycemia.
3. Clinical manifestations: Typical hypoglycemic symptoms include sympathetic excitation symptoms such as sweating, palpitation, and shaky hands, as well as symptoms of impaired brain function. However, in older patients with diabetes, hypoglycemia shows a great heterogeneity in its clinical manifestations and is usually not manifested as sympathetic excitation symptoms,^207^ but as dizziness, blurred vision, conscious disturbance, and other impaired brain function symptoms. Nocturnal hypoglycemia can be manifested as reduced sleep quality and nightmare. Clinically, physicians should be highly vigilant about atypical hypoglycemic symptoms of older patients with diabetes. Older patients with diabetes are very likely to develop severe hypoglycemia due to decreased neurological responses and a lower threshold of response to hypoglycemia. Compared to non‐elderly diabetic patients, these patients are at higher risk of asymptomatic hypoglycemia, and the risk of severe hypoglycemia and even death is higher in older patients with diabetes who have asymptomatic hypoglycemia.^208^ Repeated episodes of hypoglycemia may further decrease the neurological responses,^209^ and patients may even experience coma directly in the absence of sympathetic activation.^210^ The above conditions, if occurring in the nighttime, are extremely dangerous because it is difficult to identify and timely treat the disorders.
4. Prevention and treatment: It should be particularly highlighted that improper use of insulin and secretogogue is an important contributor to hypoglycemia in older patients with diabetes.^211^ Therefore, insulin, sulphonylurea secretogogues, glinide secretogogues, and other glucose‐lowering medications at high risk of hypoglycemia should be used with caution. If such drugs are used, blood glucose monitoring should be strengthened, and if necessary, the CGM can be applied.^212^ Single‐agent use of metformin, DDP‐4 inhibitors, alpha‐glucosidase inhibitors, GLP‐1RAs, and SGLT2 inhibitors is associated with a lower hypoglycemia risk.^213^ However, because older patients with diabetes often have multiple concurrent diseases, caution should still be exercised when using these drugs to avoid an increased risk of hypoglycemia due to potential interactions with other medications.^214^ An increased hypoglycemia risk is associated with strict glycemic control.^215^ Therefore, it is crucial to set reasonable HbA_1c_ targets tailored to individual patient circumstances in elderly diabetic patients to reduce the occurrence of hypoglycemia. When setting personalized HbA_1c_ targets, careful consideration should be given to several factors, including the duration of diabetes, the patient's life expectancy, the use of insulin and secretagogues, history of severe hypoglycemia, and the presence of other conditions (such as multiple comorbidities, cognitive impairment, polypharmacy).^216^ Older patients with T1DM have a high incidence of severe hypoglycemia, especially those with a prolonged disease course.^217^ Results from RCTs suggest that CGM may contribute to reducing the risk of hypoglycemia in elderly T1DM patients,^218^ and its use should be considered for eligible patients when necessary.

### 13.2 Hyperglycemic crisis

Hyperglycemic crises mainly include hyperglycemia hyperosmolar state (HHS) and diabetic ketoacidosis (DKA). HHS is one of the severe acute complications of diabetes, clinically characterized by severe hyperglycemia, elevated plasma osmolarity, dehydration, and altered consciousness, usually without obvious ketosis and metabolic acidosis. Older patients with diabetes are the main population affected by HHS. HHS has a higher mortality rate than DKA, approximately 10 times higher,^219^ and requires high attention from clinicians. Infection is the primary trigger for HHS, followed by inappropriate discontinuation of hypoglycemic agents such as insulin, or the presence of other concurrent diseases such as myocardial infarction, cerebrovascular events, and trauma.^220^ The onset of HHS is latent, with 30% to 40% of HHS patients never diagnosed with diabetes before. The clinical manifestations of HHS include hyperglycemic symptoms, dehydration symptoms, and neurologic symptoms. It is manifested as olydipsia, polydipsia, indifference, somnolence, and even hallucinations, epileptic seizure, and coma in patients. Due to poor skin elasticity of the elderly, the signs of dehydration are more difficult to identify. The diagnostic criteria for HHS include: plasma glucose level ≥33.3 mmol L^−1^, effective plasma osmolality ≥320 mOsm L^−1^, absence of significant metabolic acidosis, and absence of severe ketosis. In terms of treatment, fluid replacement is the crucial initial step that can help restore the blood volume and renal perfusion, improve peripheral circulation, and lower the blood glucose level. For older patients with diabetes, excessively slow and insufficient fluid replacement is more likely to cause hypotension and prerenal renal insufficiency, while excess and excessively fast fluid replacement tend to cause pulmonary edema and cardiac insufficiency. Therefore, the speed of fluid replacement should be adjusted individually according to the blood pressure, renal function, and cardiac function status. Continuous intravenous insulin injection, active potassium supplement, and close monitoring of blood potassium should be performed to avoid malignant arrhythmia caused by blood potassium decreased.

Although DKA is not common in older patients with diabetes, a variety of complications and comorbidity are more likely to occur in these patients than in non‐elderly ones, causing organ and system dysfunctions, and ultimately resulting in poor outcomes.^221^ It should be noted that co‐existence of DKA and HHS is not rare. In recent years, with the use of immune checkpoint inhibitors, diabetes onset following the use of these inhibitors is often characterized by the onset of DKA^222^ and may also be accompanied by HHS.^223^ Therefore, older patients with diabetes using immune checkpoint inhibitors should be vigilant for both DKA and HHS. Older patients with diabetes using SGLT‐2 inhibitors should also be aware of the potential occurrence of DKA.^224^ Abdominal pain, nausea, and vomiting are common clinical manifestations of DKA, with neurological symptoms being more prominent in elderly diabetes patients experiencing DKA, while gastrointestinal symptoms may be less apparent. The diagnostic points include: blood glucose increased, blood ketone bodies and /or urine ketone bodies increased, as well as blood PH value and/or carbon dioxide combining power decreased. Regardless of DKA or HHS alone, or both, the treatment should adhere to the following principles: initiate fluid replacement as soon as possible to restore the blood volume, lower blood glucose and correct electrolyte and acid–base imbalances, and meanwhile actively look for and remove the cause, prevent complications, and reduce mortality.

### 13.3 Lactic acidosis

Although rare, lactic acidosis is highly fatal and extremely dangerous. In diabetic patients, renal insufficiency may cause accumulation of biguanides in the body, increasing the risk of lactic acidosis. Older patients with diabetes who have hepatic/renal insufficiency should be alert to lactic acidosis when using biguanides.

## 14 CHAPTER 12: COMORBIDITIES OF DIABETES IN THE ELDERLY

### 14.1 Heart failure

**Key points**

1. Drugs that increase risk of heart failure should be used with caution and contraindicated in older patients with diabetes who are at high risk of heart failure or complicated with heart failure. (B)
2. SGLT‐2 inhibitors reduce the risk of hospitalization for heart failure in patients with heart failure and are preferred for older patients with diabetes and concurrent heart failure. (A)

Both age and diabetes are risk factors for heart failure, and the prevalence of heart rate is up to 22.3% in older patients with diabetes.^225^ In recent years, foreign single‐center studies have shown that the prevalence of heart failure in hospitalized elderly diabetic patients is even as high as 56%.^226^ Heart failure with preserved ejection fraction (HFpEF) is more common in elderly diabetic patients, which is prone to be underdiagnosed. Studies have shown that heart failure is missed in up to 27.7% of elderly diabetic patients.^227^ Heart failure is independently associated with cardiovascular death and hospitalization risk.^228^ Although heart failure is common in older patients with diabetes, there is lack of clinical evidence required for developing the best treatment strategy. Insulin may exacerbate heart failure by causing water–sodium retention and thereby should be used with caution in older patients with diabetes and concurrent heart failure. TZDs are contraindicated in older patients with diabetes who are rated as New York Heart Association functional class III or higher. In the SAVOR study, the rate of hospitalization for heart failure as one of the components of the secondary endpoints was elevated in the saxagliptin‐treated group compared to the placebo group,^229^ and saxagliptin should be used with caution when selecting a DPP‐4 inhibitor for older patients with diabetes with heart failure. Metformin is safe and beneficial in patients with diabetes and heart failure^230^, ^231^ and should be retained in the treatment regimen if there are no contraindications or intolerance. SGLT‐2 inhibitors can reduce the risk of hospitalization for heart failure, and the results were similar in the elderly subgroup to the overall population.^73^ This class of drug should be preferred in elderly diabetic patients with comorbid heart failure.^232^ Cohort studies have shown that GLP‐1RAs also improve composite endpoints including hospitalization for heart failure compared to DPP‐4 inhibitors.^233^

### 14.2 Osteoporosis

**Key points**

1. Bone mineral density (BMD) measurement by dual‐energy X‐ray absorptiometry and the fracture risk assessment tool FRAX suggests the fracture risk of older patients with diabetes but may underestimate such risk. (B)
2. Older patients with diabetes and comorbid osteoporosis should be cautious about using medications that may increase the risk of osteoporosis or fractures. (B)

Osteoporosis is an aging‐related disease, with a significantly higher prevalence of osteoporosis in people aged 60 years and above, and the prevalence of vertebral fractures in women aged 80 years and above can be as high as 36.6%.^234^ The risk of fracture in patients with diabete significantly exceeds that of the non‐diabetic population.^235^ Data from Beijing showed that the prevalence of diabetes with osteoporosis was 13.4% to 14.8% from 2016 to 2018, and the prevalence of osteoporosis in older patients with diabetes was higher than that in young patients with diabetes.^236^ Therefore, older patients with diabetes are a high‐risk group for osteoporotic fractures, and once fractures occur in older adults with diabetes, the quality of life is seriously affected, with high rates of disability and mortality. Fall history, fracture history, low grip strength, and elevated HbA_1c_ are risk factors for fractures in older T2DM patients.^237^ BMD measurement by dual‐energy X‐ray absorptiometry and the fracture risk assessment tool FRAX can be used to assess the fracture risk of diabetic patients but may underestimate such risk. T2DM is more prone to fracture than non‐diabetic population at the same BMD.^238^ The fracture risk assessment tool, FRAX, similarly underestimates fracture risk in diabetic patients.^239^ Education on osteoporosis prevention and treatment in older patients with diabetes should be strengthened, with active assessment of fracture risk and early intervention. Older patients with diabetes with osteoporosis should be cautious about using glucose‐lowering medications that may increase the risk of osteoporosis or fractures. The Chinese expert consensus recommends that the management of diabetes mellitus combined with osteoporosis refer to non‐diabetic patients.^240^ Multi‐disease coexistence and multiple medications need to be considered in the selection of osteoporosis medications, and individualized medications and monitoring should be performed after a thorough assessment and weighing of the pros and cons.

### 14.3 Sarcopenia and frailty

**Key points**

1. Older patients with diabetes should be actively assessed for geriatric syndrome. (B)
2. The high prevalence of sarcopenia in older patients with diabetes is an important cause of frailty in these patients. (A)
3. All older patients with diabetes should be assessed for sarcopenia and frailty, so that early intervention can be given to improve their prognosis. (B)

Geriatric syndromes are a cluster of multiple abnormalities prevalent among older adults, which severely affect their quality of life and lead to poor clinical outcomes. The Asia‐Pacific consensus states that geriatric syndromes should include Alzheimer's disease, pressure sores, hearing loss, visual loss, sarcopenia, frailty, and fall, etc.^241^ Compared to non‐diabetic older people, older patients with diabetes have a higher prevalence of geriatric syndromes.

Sarcopenia is an aging‐related disease with a prevalence of 14.8% in older patients with diabetes in China.^242^ T2DM is associated with decreased muscle strength and poor muscle mass, exacerbating age‐related sarcopenia.^243^ T1DM can also develop sarcopenia,^244^ the pathogenesis of which is related to autoimmune damage.^245^ Diabetic patients with sarcopenia have more severe abnormalities of glucose metabolism, poorer nutritional status, and are also more likely to have comorbid osteoporosis and falls.^246^ Sarcopenia makes it more difficult for older patients with diabetes to control their blood glucose, reduces the ADLs, and increases mortality.^247^ Therefore, sarcopenia in geriatric diabetes should be emphasized. Due to changes in body composition associated with increased age, older patients with diabetes can develop both obesity and sarcopenia, that is, sarcopenic obesity.^248^ The prevalence of sarcopenic obesity increases with age, reaching 48.0% in women and 27.5% in men over 80 years of age.^249^ Sarcopenia in obese older patients with diabetes should not be ignored to avoid mindless weight loss and medication‐induced underweight. It is recommended to assess older patients with diabetes for sarcopenia according to the screening and diagnostic criteria of the Asian Working Group for Sarcopenia.^250^ In the community primary care setting, screening can be done by means of scales (SARC‐F scale or SARC‐F + Calf Circumference Scale), assess muscle strength and physical function, and lifestyle interventions can be considered for the possibility of sarcopenia. In addition to the screening and assessment tools above, the diagnosis of sarcopenia can be further clarified at a medical facility or clinical research center by determining the amount of skeletal muscle in the extremities.

Frailty refers to a pre‐disability state in which the body becomes more and more vulnerable with age due to degenerative changes, physiological reserve function decline and various chronic diseases, and subsequently loses its ability to resist physical or mental stress. Therefore, unlike disability and co‐morbidity, the cause of frailty is somewhat reversible, and the prognosis of older people with frailty can be improved by early identification and intervention. In the elderly, diabetes increases the risk of frailty fivefold,^251^ leading to reduced mobility, increased difficulty in monitoring and managing blood glucose, and affecting patient prognosis. A meta‐analysis showed that the prevalence of frailty and pre‐frailty among community‐dwelling older patients with diabetes was 20.1% and 49.1%, respectively.^252^ The degree of frailty of the patient needs to be taken into account when setting goals for glycemic control, and simplification, switching, or downgrading of treatment regimens may be necessary in older diabetic patients with comorbid frailty, with particular consideration given to the reduction of treatments that may induce hypoglycemia, such as sulfonylureas and insulin.^253^ Meta‐analysis revealed that the use of sulfonylureas in older adults with T2DM may increase the risk of fracture, which may be linked to their triggering of hypoglycemia.^254^

Concerns and assessment of sarcopenia and frailty in older patients with diabetes and making appropriate interventions can help to improve the prognosis and reduce health care expenditures.^255^, ^256^

### 14.4 Falls

**Key points**

1. In view of the high prevalence of falls in older patients with diabetes, they should be assessed for fall risk to identify the risk factors for falls as early as possible and give early intervention. (C)
2. Fall prevention reduces fractures in older patients with diabetes. (C)

Falls are the leading cause of traumatic fractures and injury‐related deaths in Chinese elderly population. As people age, they are at increased risk of falls due to the decline in various physiological functions, including decreased gait coordination and balance resulting from functional decline in the maintenance of the musculoskeletal motor system, as well as declines in vision, hearing, vestibular function, and proprioception. Diabetes increases the risk of falls in the elderly, especially those on insulin, where the risk of falls is 94% higher than in non‐diabetic elderly.^257^ Falls, while high among older patients with diabetes, are preventable. All older patients with diabetes need to be assessed for fall risk, including past medical history, somatic functional status, environment, and psychological assessment. Major risk factors for falls in older patients with diabetes include hypoglycemia and glucose fluctuations, central and peripheral neuropathy, vascular factors, orthostatic hypotension, postprandial hypotension, diabetic ophthalmopathy, medications (antihypertensives, diuretics, sedative‐hypnotics, etc.), and sarcopenia and frailty. For older patients with diabetes who have the above‐mentioned conditions, physicians should provide active intervention and seek assistance from relevant departments to reduce the risk of fracture and fracture‐related complications in such patients. Be cautious of falls in older patients with diabetes with comorbid osteoporosis.

### 14.5 Cognitive dysfunction

**Key points**

1. Hypoglycemia increases the dementia risk of patients, while diabetic patients with cognitive disorder are more likely to experience hypoglycemic events. (B)
2. Cognitive dysfunction is of particular importance in older patients with diabetes, and it is recommended to conduct annual screening to facilitate early identification of cognitive dysfunction and dementia in such patients. (B)
3. For older patients with diabetes with cognitive dysfunction, less stringent glycemic targets should be implemented. (C)

The prevalence of cognitive dysfunction among older patients with diabetes in China is 48%, with higher prevalence among women, older age, lower education level, lower income, no spouse, and living alone.^258^ There is a bidirectional relationship between cognitive dysfunction and risk of hypoglycemia.^259^, ^260^ Hypoglycemia increases the risk of developing dementia, including vascular dementia and Alzheimer's disease. On the other hand, diabetics with cognitive dysfunction are prone to hypoglycemia. Cognitive dysfunction makes it difficult for older patients with diabetes to perform complex self‐management tasks,^261^ such as monitoring blood glucose and adjusting insulin dosage, and they also affect patients' eating times and meal rationalization. Early recognition of cognitive dysfunction is important in the management of diabetes in the elderly. Annual screening is recommended for older patients with diabetes for early detection of mild cognitive dysfunction or dementia. Simple and easy‐to‐use assessment tools are available to screen for cognitive impairment, such as the Mini‐Mental State Examination (MMSE)^262^ and the Montreal Cognitive Assessment Scale (MoCA).^263^ Modify the treatment regimen for diabetic patients with cognitive dysfunction by selecting medications with a low risk of hypoglycemia, while simplifying the treatment regimen and “de‐intensifying therapy” if possible, with less stringent glycemic targets to avoid hypoglycemic and hyperglycemic crises. Metformin and GLP‐1RAs may help improve cognitive function. A retrospective study showed that metformin was significantly associated with a reduced risk of dementia and showed a dose‐dependent effect in older patients with T2DM.^264^ GLP‐1RAs are associated with a reduced incidence of Alzheimer's disease in elderly patients.^265^ The effects of sulfonylureas, TZDs, DPP‐4 inhibitors, and SGLT‐2 inhibitors on cognitive function are unclear.^266^ Exercise also helps to improve cognitive function, and a randomized clinical trial found that tai chi was more effective than fitness walking in improving cognitive function in Chinese older patients with T2DM.^267^

### 14.6 Mental illness

**Key points**

1. The mental status of older patients with diabetes, as well as early recognition of mental illness and intervention should be concerned. (B)
2. Improvement of mental illness contributes to glycemic control and improved quality of life in older patients with diabetes. (B)

The risk of depression is higher in older patients with diabetes than in non‐diabetic older adults^268^ but is easily overlooked. Depression and anxiety may lead to less adherence in older patients with diabetes and less effective glycemic control.^269^ Attention should be paid to the mental status of older patients with diabetes for early identification of psychiatric disorders and intervention. Simplified screening tools such as the Geriatric Depression Scale can be used for early identification. A multidisciplinary collaborative care pattern can be established and perfected to improve patients' depression and anxiety symptoms, which will not only improve the quality of life, but also facilitate glycemic control. Delirium is often seen in the elderly population.^270^ If delirium occurs in older patients with diabetes, timely identification and removal of triggers, active encouragement of family members to participate in the process of non‐pharmacological treatment, and nursing support are helping to prevent and treat delirium‐related complications.

### 14.7 Hypotension

**Key points**

1. Anti‐hypertensive medications that increase the risk of orthostatic hypotension should be avoided in older patients with diabetes with orthostatic hypotension. (B)
2. Alpha‐glucosidase inhibitors may be considered in older patients with diabetes with postprandial hypotension. (B)

Aging leads to changes in cardiovascular structure and function; therefore, older adults are prone to orthostatic and postprandial hypotension.^271^ Hypotension increases the risk of falls^272^ and even increases the risk of cardiovascular events and death.^273^, ^274^ Older patients with diabetes are at higher risk of orthostatic hypotension than non‐diabetic elderly, and 40% of orthostatic hypotension patients have comorbid diabetes.^275^ Older patients with diabetes with hypertension may have concomitant orthostatic hypotension; therefore, ARBs or calcium channel blockers should be preferred in this group of patients,^276^, ^277^ and antihypertensive medications such as tamsulosin and carvedilol, which may worsen orthostatic hypotension, should be avoided.

In the elderly, intake of carbohydrate‐rich hot food and upright posture are both associated with symptomatic postprandial hypotension.^278^ The association of symptoms such as dizziness and syncope with eating and postural changes should be addressed in older diabetic patients. Lifestyle modifications may reduce symptomatic hypotension in older patients with diabetes. In addition, α‐glucosidase inhibitors help to improve the symptoms of postprandial hypotension in older patients with diabetes^50^, ^279^ and should be considered when selecting hypoglycemic agents. SGLT‐2 inhibitors have a mild blood pressure lowering effect and should be of concern in patients applying this class of hypoglycemic agents.

### 14.8 Tumors

**Key points**

1. It is recommended that older patients with diabetes should be screened for tumors that match their age and gender. (B)
2. New‐onset older patients with diabetes need to be alerted to the possibility of pancreatic cancer. (B)

Patients with diabetes have an increased risk of tumors, including hepatocellular carcinoma, hepatobiliary carcinoma, pancreatic cancer, breast cancer, ovarian cancer, endometrial cancer, and gastrointestinal malignancies.^280^, ^281^ It is recommended that older patients with diabetes get tumor screening matched to their age and gender. As elevated blood glucose is sometimes the first clinical manifestation of pancreatic cancer,^17^ new‐onset older patients with diabetes should be screened to exclude the possibility of pancreatic cancer. Some hypoglycemic agents may have a role in reducing tumor risk, including metformin^282^ and DPP‐4 inhibitors,^283^ etc.

### 14.9 Obstructive sleep apnea syndrome (OSAS)

**Key points**

1. OSAS is associated with glycemic variability and diabetic complications. (B)
2. It is recommended that obese older patients with diabetes be routinely screened for OSAS. (B)
3. Weight reduction and continuous positive airway pressure (CPAP) are effective treatments for OSAS with improved glycemic control. (B)

OSAS, one of the common types of sleep disorders, is typified by pathologic sleep fragmentation and is a respiratory sleep disorder that results in abnormal glucose metabolism, secondary hypertension, and multiple organ damage. OSAS leads to increased secretion of multiple stress hormones, aggravated glycemic variability, and increased difficulty in glycemic control. The prevalence of OSAS increases with age,^284^ and obesity is also an important risk factor for OSAS. OSAS is usually more severe in those with diabetes^285^ and closely associated with the occurrence and progression of diabetic complications. The prevalence of OSAS is higher in men than in women, and obviously higher in postmenopausal women than in premenopausal ones. It is recommended that obese older patients with diabetes be routinely screened for OSAS.

For older patients with diabetes with OSAS, active lifestyle interventions for weight loss, smoking cessation, alcohol restriction, and avoiding strong tea and coffee, as well as daytime overexertion and excitement that affects sleep, should be carried out.^286^ CPAP, an effective treatment for OSAS, can significantly improve the blood glucose level and insulin resistance of diabetic patients.^287^, ^288^ Drugs that can lead to weight gain should be always avoided, and biguanides should be used with caution or contraindicated in patients with hypoxemia. In addition, attention should be paid to excluding and treating underlying diseases that can cause or exacerbate OSAS, such as hypothyroidism.^289^

### 14.10 Sleep disorder

**Key points**

1. Older patients with diabetes have a variety of factors that can lead to sleep disturbances and should be concerned about their sleep. (B)
2. Intervention for sleep disorder can help improve the quality of sleep and life in older patients with diabetes, as well as improve blood glucose. (C)

In addition to OSAS, older patients with diabetes may also have various forms of sleep disorder. This section discusses sleep disorders not due to OSAS. Elderly people are characterized by physiological deterioration of brain function, susceptibility to anxiety, and weaker psychological tolerance, making them a high‐risk group of sleep disorders.^290^ Nocturia, painful neuropathy, somatic symptoms, and mental disorders in diabetic patients may lead to sleep disorders. In older patients with diabetes, the risk of sleep disorders is high, affecting glycemic control and quality of life, and should be paid attention to. Aggressive health education and intervention for older patients with diabetes with sleep disorders can help improve sleep and quality of life, as well as improve blood glucose.^291^

### 14.11 Oral disorder

**Key points**

1. Periodontitis is highly prevalent in older patients with diabetes, which increases the difficulty of glycemic control. (B)
2. Encourage older patients with diabetes to develop good hygiene habits and have regular oral examinations. (B)
3. Good glycemic control is conducive to the treatment of oral disorder, and in turn, the control of oral disorder is beneficial for the improvement of blood glucose. (B)

The risk of oral disorder increases with age. Oral disease can occur in older adults for a variety of reasons, including dry mouth, decreased oral motor function, and decreased ability to manage oral hygiene. The prevalence, course, and severity of oral diseases are significantly increased in diabetic patients compared to non‐diabetic population.^292^ Periodontitis is one of the common concomitant diseases of diabetes, and diabetes is an important risk factor for periodontitis. Diabetic patients have a nearly threefold increased risk of periodontitis compared to the non‐diabetic population. Compared to young diabetic patients, older patients with diabetes face more prevalent and severe oral problems such as dental caries, xerostomia, and periodontal disease. Good glycemic control facilitates the treatment of oral lesions such as periodontitis, and treatment for oral lesions such as periodontitis favors improved glycemic control.^293^, ^294^, ^295^ Encourage older patients with diabetes to develop good hygiene habits: adhere to effective daily brushing; remove tartar, food impaction, and other local irritants; keep the oral environment clean; and conduct regular oral examination and periodontal scaling.

### 14.12 Skin problems

**Key points**

1. Focus on skin problems in older patients with diabetes and actively treat diabetes‐induced skin lesions. (B).
2. Poor glycemic control can exacerbate skin lesions, and skin lesions can lead to elevated blood glucose. (C).

Patients with diabetes mellitus can develop a variety of skin lesions, including: (1) skin infections: bacterial, fungal, or viral infections; (2) skin diseases directly related to diabetes mellitus: diabetic maculopapular, acanthosis nigricans, etc.; (3) the skin manifestations of chronic complications of diabetes mellitus: skin ulcers or even gangrene caused by diabetic foot; (4) the skin changes due to diabetes mellitus treatment: for example, subcutaneous fat proliferation, subcutaneous fat reduction, etc., caused by the injection of insulin.

Itchy skin is a common symptom in diabetic patients, with 12.7% of T2DM patients experiencing itchy skin, and the incidence of itchy skin is even higher in T2DM with comorbid diabetic peripheral neuropathy (20.5%).^296^ Advanced age and long disease duration are also risk factors for pruritus.^297^ Thus, the incidence of pruritus in older patients with diabetes is high, and pruritus seriously affects the life treatment of older patients with diabetes. Reducing bathing times and applying moisturizers can improve dry skin and may help relieve itching. Poor glycemic control in diabetic patients can lead to an increased risk of skin lesions, and skin lesions, such as skin infections, can also lead to elevated blood glucose. Endocrine specialists should be concerned about the skin conditions of older patients with diabetes and manage them aggressively, referring them to dermatologists for further consultation if necessary.

## 15 CHAPTER 13: POLYPHARMACY IN OLDER PATIENTS WITH DIABETES

**Key points**

1. Polypharmacy is common and inevitable in older patients with diabetes. (B)
2. During the choice of glucose‐lowering medications, full consideration should be given to drug–drug interactions, to avoid adverse reactions. (B)

Polypharmacy refers to the concurrent use of greater than or equal to five medications in a single individual. Older patients with diabetes are usually comorbid with hypertension, coronary heart disease, stroke, and chronic respiratory diseases, making polypharmacy common and inevitable among them. Polypharmacy increases the risk of drug interaction and thereby has the potential to jeopardize the glucose‐lowering efficacy for older patients with diabetes and increase the risk of hypoglycemia. Sulphonylureas are mainly metabolized by the hepatic CYP2C9 enzyme. Concurrent use of fluconazole, cimetidine, and other CYP2C9 inhibitors in older patients will slow down the metabolism of sulphonylureas to increase the hypoglycemia risk. Concurrent use of acarbose with warfarin will cause prothrombin time (PT) and/or international normalized ratio (INR) increase and raise the bleeding risk, thereby requiring timely dose adjustment. Repaglinide mainly metabolized by CYP2C8 and CYP3A4 may have an enhanced and/or prolonged glucose‐lowering effect and an increased hypoglycemia risk when used in combination with clopidogrel, ketoconazole, ACEIs, and monoamine oxidase inhibitors, etc. Saxagliptin is metabolized by CYP3A4, and when used in combination with strong CYP3A4 inhibitors such as ketoconazole, itraconazole, atazanavir, ritonavir, clarithromycin, and telithromycin, it significantly increases plasma drug levels, necessitating a reduction in dosage; co‐administration with carbamazepine accelerates its metabolism, significantly reducing its hypoglycemic effect.^298^ Vildagliptin used in combination with ACEIs may increase the risk of angioneurotic edema.^299^ Rosiglitazone and pioglitazone are metabolized by CYP2C8, and drugs like gemfibrozil and clopidogrel slow down their metabolism, increasing plasma drug levels^300^; inducers of CYP2C8 such as rifampin accelerate drug metabolism, reducing their efficacy. Therefore, the drug use profile of older patients should be a factor to consider when developing the drug therapy regimen, and for patients with polypharmacy, glucose‐lowering medications with less drug interactions should be chosen to avoid drug–drug interactions.

## 16 CHAPTER 14: SPECIAL CIRCUMSTANCES IN THE OLDER PATIENTS OF DIABETES

**Key points**

1. For older inpatients with diabetes whose CGA results are Group 1 or Group 2, the FPG goal is from 6.1 to 7.8 mmol L^−1^ and postprandial 2‐h PG or RPG goal is from 7.8 to 10.0 mmol L^−1^, and these goals can be appropriately relaxed for patients of Group 3. (B)
2. Older patients with diabetes who plan to receive elective surgery should have their blood glucose controlled between 7.8 and 10.0 mmol L^−1^ in the perioperative period. For these patients undergoing emergency surgery, it is not suggested to set too stringent glycemic goals before the surgery. (B)
3. Older patients with diabetes are prone to infections that will make blood glucose difficult to be control, and even cause hyperglycemic crisis. (B)
4. For patients in their late life, the primary treatment goal is to maintain dignity, reduce pain, and guarantee quality of life; blood glucose, blood pressure, and blood lipid control goals should be approximately relaxed to reduce the intensity of treatment, but attention should be paid to avoiding diabetic emergencies. (B)

### 16.1 Hospital management of diabetes in the older patients

Older patients with diabetes are often hospitalized for various problems unrelated to diabetes, such as cardiovascular diseases, dyspnea, and infections. Endocrinologists should work with other specialists to manage the blood glucose of such patients. Conditions of inpatients may change within a short time, requiring timely identification and treatment regimen adjustment from physicians. In case of changes in patients' blood glucose levels due to application of glucocorticoid therapy, nasal feeding, total parenteral nutrition (TPN), and dialysis, the glucose‐lowering regimen should be re‐assessed in a timely manner. In addition, clinicians should always take a patient's age, life expectancy, overall health status, functional status, and cognitive ability into account, and weigh glycemic control and adverse reactions. During hospitalization in a department of other disciplines other than endocrinology, clear glycemic goals are conducive to glycemic control.^301^ According to the suggestions in Chinese Expert Consensus on the Blood Glucose Management of Inpatients,^302^ older inpatients with diabetes whose CGA is Group 1 or Group 2 should adopt general glycemic goals: FPG between 6.1 and 7.8 mmol L^−1^, 2‐h PG or RPG between 7.8 and 10.0 mmol L^−1^; for those who have a high risk of hypoglycemia, a short life expectancy, and CGA of Group 3, the goals can be approximately relaxed to FPG between 7.8 and 10.0 mmol L^−1^ and 2‐h PG or RPG between 7.8 and 13.9 mmol L^−1^ based on the patient's individual condition, but hyperglycemic crisis should be avoided.

### 16.2 Nursing home management of diabetes in the older patients

With the accelerated progress of the aging of population in China, the health management of older patients with diabetes in nursing home has become increasingly important. For nursing homes with HCPs, older patients with diabetes can be managed by referring to hospital management mentioned above. However, at present, nursing homes in China mainly provide basic life care, lacking health management services for chronic diseases such as diabetes. It is necessary to strengthen education on diabetes for caregivers.^136^ Overly stringent dietary control provides limited help for the glycemic control of older patients with diabetes^303^ and may increase the risk of malnutrition. In setting glycemic management goals, it is crucial to fully consider the complications and comorbidities, functional status, and life expectancy of the older patients.^304^ Dietary and exercise plans should be formulated according to different overall health status, glycemic control, and complication status. Currently, the portable glucosemeters are commonly employed in nursing homes HbA_1c_ testing and continuous glucose monitoring (CGM) may also be conducted in some nursing homes. For older patients treated with sulfonylurea drugs or insulin, the use of CGM to assess the risk of hypoglycemia may be considered.^136^ It is imperative to promptly identify acute diabetic complications in older patients; and suspected cases of diabetic ketoacidosis (DKA) and/or hyperosmolar hyperglycemic state (HHS) should be urgently managed and immediately transferred.^304^ Attention should be given to foot care in older patients with diabetes, and those with any skin ulceration of the feet should be referred promptly to an endocrinologist or a department related to diabetic foot disorders.

### 16.3 Home management of diabetes in the older patients

Diabetes is highly prevalent in the elderly living at home,^305^ being one of the chronic diseases requiring great concern from them. The home management of older patients with diabetes should include the self‐management of the patients living at home and their family member's care, as well as chronic disease management carried out by community health care institutions. The multidisciplinary team intervention pattern can optimize the blood glucose and mindset of these patients and increase their self‐management ability to a certain extent.^306^ The home management of older patients with diabetes should pay attention to preventing acute events such as hypoglycemia, hyperglycemic crisis, and falls, etc.

### 16.4 Perioperative management

The perioperative glycemic goal of older patients with diabetes is from 7.8 to 10.0 mmol L^−1^.^108^, ^307^ For older patients with severe comorbidities and frequent events of hypoglycemia, the control target can be as high as 13.9 mmol L^−1^.^307^ For patients who plan to receive cardiac operation or other sophisticated surgeries, it is suggested to consider a more stringent glycemic goal, i.e., from 6.1 to 7.8 mmol L^−1^, with the hypoglycemia risk considered.^308^ To minimize the impact of blood glucose, patients with diabetes are preferably operated on in the early morning or as early as possible. Patients with later surgery times should have their blood glucose continuously monitored in the ward.^309^ Fasting is required for surgery, all oral glucose‐lowering medications (OGLMs) should be discontinued on the morning of the operation day, and blood glucose monitoring should be carried out once every 4–6 hours during the fasting period. During the surgery, the frequency of blood glucose monitoring should be increased according to the patient's condition; when the blood glucose level is beyond the control goal, short‐acting human insulin or rapid‐acting insulin analogue should be given. For patients undergoing minor surgery whose blood glucose can be controlled well by OGLMs, it is not necessary to use insulin during the surgery, but their OGLMs should be resumed after surgery. During fasting, attention should be paid to discontinuing medications with a high risk of hypoglycemia, and the use of SGLT‐2 inhibitors should be suspended during surgery to avoid the risk of DKA.^309^ For patients undergoing major and intermediate surgeries, especially those with suboptimal glycemic control, it should be switched to insulin therapy in a timely manner, and during the surgery, insulin therapy should be continued with close monitoring of blood glucose. Intravenous infusion of insulin may be given to control blood glucose before restoration of normal diet after surgery, and switched to subcutaneous injection after normal diet is restored. For patients who plan to receive an emergency surgery, too stringent glycemic goals should be avoided before surgery, pre‐procedure preparation should be completed as soon as possible, and their blood glucose should be lowered by intravenous infusion of insulin and monitored during the surgery. During the perioperative period, attention should be paid to monitoring fluctuations of blood glucose and watching out for hypoglycemia.

### 16.5 Infection and vaccination

Patients with diabetes are susceptible to concurrent infections with bacteria, fungi, viruses, and atypical pathogens, with the common infection sites including the respiratory system, urinary system, skin, and soft tissues. Compared to the general population, patients with T2DM have a worse prognosis upon infection, with higher rates of incidence and mortality from sepsis.^310^ Patients with diabetes are at a greater risk of skin and soft tissue infections (SSTIs) than non‐diabetic patients, yet patients with severe diabetic neuropathy and vasculopathy may not exhibit typical SSTI symptoms.^311^ Infections are highly prevalent and not easy to be controlled in older patients with diabetes.^312^ A retrospective cohort study showed that compared to patients with diabetes aged 40 to 49 years, the risk of hospitalization was 3 to 4 times higher in patients aged 80 to 89 years, with a high proportion of infection‐related hospitalizations.^313^ Concurrent infections in older patients who have a long course of disease and suboptimal glycemic control will make blood glucose more difficult to control and may even induce hyperglycemic crisis.

Coronavirus disease 2019 (COVID‐19) is associated with hyperglycemia in both patients with known diabetes and individuals not previously diagnosed with diabetes. A study from Wuhan on hospitalized patients (mainly the elderly with COVID‐19) indicated that 21.6% had a history of diabetes, 20.8% were newly diagnosed with diabetes, and 28.4% were newly diagnosed with abnormal blood glucose levels based on initial blood sugar measurements upon admission. Blood glucose levels at the time of hospital admission in patients with diabetes were associated with an increased risk of all‐cause mortality. Patients with newly diagnosed diabetes were more likely to be admitted to intensive care units and invasive mechanical ventilation and had higher prevalence rates of acute respiratory distress syndrome, acute kidney injury, or shock, with the longest hospital stays.^314^ The exact mechanisms behind new‐onset diabetes in COVID‐19 patients remain unclear, with possible causes including previously undiagnosed diabetes, stress hyperglycemia, viral infection, or steroid‐induced diabetes.^315^ Obesity, hyperglycemia, and cardiovascular as well as renal diseases are risk factors of poor prognosis in COVID‐19 patients. Therefore, for the long‐term management of acute COVID‐19 infections and persistent symptoms (i.e., long COVID) in patients with diabetes, the use of glucose‐lowering medications that do not increase body weight and improve cardiorenal outcomes (such as SGLT‐2 inhibitors and GLP‐1RAs) is recommended, although there is no evidence from long‐term follow‐up.^315^

Good glycemic control enhanced personal hygiene management, and necessary vaccination can prevent serious infections to a certain extent. Older patients with diabetes should receive influenza and pneumococcal vaccines based on individual conditions to reduce their hospitalization and death risks. However, the live attenuated vaccines should be avoided for the influenza vaccination.^316^ For older patients with diabetes comorbid with infection, stringent glycemic control is the primary measure; however, for those with multiple concurrent diseases and a short life expectancy, the glycemic goals can be approximately relaxed while effective anti‐infective therapy is given, and surgical therapy should be administered as needed.

### 16.6 Palliative care

Special management different from other periods should be carried out for older patients with diabetes who enter their end stage of life. In this stage, maintaining a patient's dignity, reducing pain, and guaranteeing quality of life are of vital importance. Patients have the right to reject examinations and treatment; HCPs should consider reducing diagnostic examinations and unnecessary treatment. Whenever possible, the HCPs, the patient, and his/her family should all participate in the medical decision‐making process. In this stage, stringent management of blood glucose, blood pressure, and blood lipids are not the primary goal; palliative care with the aim of reduction in the frequency of blood glucose monitoring, prevention of hypoglycemia and hyperglycemic crisis, and alleviation of patient's pain and other discomforts should be carried out to improve patient's quality of life. With respect to the choice of glucose‐lowering regimen, OGLMs can be used as first‐line medications, but it is not suggested to use drugs associated with gastrointestinal adverse reactions; if necessary, basal insulin can be given. Patients with T2DM and organ damage should decrease the dosage of medications that carry a risk of hypoglycemia, allowing blood glucose levels to remain at the upper limit of normal range or within the acceptable range in which acute metabolic abnormalities are absent. Medication can be discontinued when necessary, taking the amount of food intake into account. In the case of patients with organ failure, the insulin dosage for T1DM patients can be reduced in correspondence with decreased food intake but should not be completely discontinued. There is no definitive consensus on the treatment approach for T1DM patients in a terminal state; however, low dose of basal insulin may be used to prevent acute complications from hyperglycemia.^136^

## 17 CHAPTER 15: T1DM AND RELATED PROBLEMS IN THE OLDER PATIENTS

**Key points**

1. With the improvement in medical care, the number of older patients with T1DM will increase significantly. (C)
2. Glycemic goals and treatment strategy should be individualized for older patients with T1DM. (C)
3. For older T1DM patients with cognitive decline or decline in physical function, the treatment strategy should be as simple as possible, and meanwhile nursing support should be strengthened. (C)
4. For older patients with T1DM, the use of CGM or insulin pumps can improve glycemic control and reduce the incidence of hypoglycemia (B).

Older patients with T1DM refer to T1DM patients aged 65 years or older, including those diagnosed before, at, and after the age of 65 years. With the improvement of people's living standards and medical care, the life span of T1DM patients is prolonged, and the number of older patients with T1DM will increase significantly. The prevalence of T1DM in the elderly is relatively low, yet latent autoimmune diabetes in adults (LADA) is not uncommon in this age group. Compared with younger‐onset patients, elderly‐onset LADA patients have better residual pancreatic β‐cell function, more pronounced insulin resistance, and more pronounced characteristics of metabolic syndrome. The proportion of positive pancreatic autoantibodies in elderly‐onset LADA patients is similar to that in younger‐onset LADA patients, but there is a significant difference in the HLA‐DQ genetic background between the two groups.^317^

Similar to the management of T2DM in the elderly, glycemic goals and glucose‐lowering regimen should be developed for older patients with T1DM based on their age, life expectancy, functional status, underlying diseases, and complication profile, etc. Due to difficulties in blood glucose management, the risks of hyperglycemic crisis and hypoglycemia are higher in older patients with T1DM than in older patients with T2DM. Before the occurrence of microvascular or macrovascular complications, more stringent glycemic goals may be considered for older patients with T1DM, but the hypoglycemia risk must be weighed. Long course of disease is a risk factor for hypoglycemia in older patients with T1DM, and the incidence of severe hypoglycemia is up to 18.6% in older patients with T1DM >40 years,^217^ for whom the glycemic goals should be appropriately relaxed.

In recent years, there have been significant advancements in diabetes management technology, particularly in the fields of insulin injection and blood glucose monitoring. Studies focusing on older patients with T1DM have shown that those using CGM or insulin pumps have lower levels of HbA_1c_, fewer hypoglycemic events, and less glycemic variability.^318^ The WISDM randomized clinical trial indicated that CGM used in older patients with T1DM can reduce the occurrence of hypoglycemia and improve overall glycemic control compared to fingerstick blood glucose monitoring.^319^ Furthermore, research has confirmed the safety of hybrid closed‐loop insulin pumps in older patients with T1DM. Compared with the sensor‐augmented pump (SAP) group, the hybrid closed‐loop group had higher Time‐in‐Range (TIR) and lower Time‐Below‐Range (TBR).^320^ It should be noted that the aforementioned clinical trials have included few participants aged 70 and older.

Due to the complexity of disease management, the self‐management process of older patients with T1DM depends on their good cognitive ability. Older T1DM patients with severe hypoglycemia are more likely to have cognitive decline; therefore their insulin therapy regimen should be simplified. T1DM may cause decline in physical function of the elderly, which results in decreased self‐management ability of older patients with T1DM; therefore, nursing support should be strengthened. Older patients with T1DM who are unable to live independently require assistance from nursing personnel in eating, activities, and injection of insulin.

There is a relative lack of evidence‐based medical proof for blood pressure, blood lipid, and proteinuria management in older patients with T1DM. Clinicians should determine the treatment goal and strategy based on the individual condition of each patient.

## 18 CHAPTER 16: DIABETES MANAGEMENT–RELATED TECHNOLOGIES

**Key points**

1. HbA_1c_ should be tested once every 3 months before the goal is achieved and once every 6 to 12 months after the goal is achieved; for patients on insulin therapy or at high risk of hypoglycemia, self‐monitoring of blood glucose is required, and patients whose condition (especially those with T1DM) permit can wear continuous glucose monitoring. (B)
2. Time in range is a new supplementary target for evaluating patients' glycemic control level. (B)
3. The insulin injection method should be correctly mastered and attention should be paid to induration, adipose hyperplasia, and atrophy caused by the injection therapy. (B)
4. Insulin pump may improve glycemic control, but it is necessary to educate patients and their families on knowledge about insulin pumps, so that insulin pump–related complications can be avoided. (B)

### 18.1 Blood glucose monitoring

Blood glucose monitoring techniques include self‐monitoring of blood glucose, CGM, glycated albumin, and HbA_1c_, etc.

HbA_1c_ is clinically used as the gold standard for evaluating long‐term glycemic control and an important basis for adjustment of treatment regimen. For patients who have not achieved the HbA_1c_ goal, it is suggested to test HbA_1c_ once every 3 months. Once the goal is achieved, HbA_1c_ can be tested once every 6 to 12 months. However, HbA_1c_ also has certain limitations; for example, it cannot reflect blood sugar fluctuations or capture hypoglycemic events. It is suggested that older patients with diabetes on insulin therapy should carry out self‐monitoring of blood glucose primarily while fasting and before meals, to find out their baseline blood glucose levels; for those at risk of nocturnal hypoglycemia, it is suggested to monitor bedtime and nighttime blood glucose levels additionally based on the patient's condition; however, for those whose blood glucose is controlled stably by OGLMs, frequent monitoring is not required.

In older patients with diabetes, CGM can further improve HbA_1c_ and meanwhile reduce glycemic variability, without increasing the hypoglycemia risk.^321^, ^322^ Due to poor pancreatic islet function and great glycemic variability, T1DM patients are more likely to benefit from CGM. It is suggested that clinicians make suggestions on whether to wear CGM after comprehensive assessments based on the patient's blood glucose condition, cognitive level, mobility, economic condition, etc. Among older T2DM patients using basal insulin, those using CGM show an increase in TIR compared to patients using blood glucose monitoring, with benefits consistent with younger patients.^323^

TIR refers to the length of time in 24 h that glucose is in the target range or the percentage of the target range. Normally, the blood glucose range of TIR in adult patients with diabetes is defined as 3.9–10.0 mmol L^−1^, but there is still lack of studies on TIR in older patients with diabetes. In recent years, international consensus recommends that TIR can be used as a measure for evaluating the glycemic control condition of adult patients with T1DM and T2DM, and TBR and TAR can also be used as parameters for evaluating the efficacy of treatment regimen. Studies in China have shown that, in T2DM patients, TIR is independent of HbA_1c_ but associated with microvascular complications of diabetes (mean age of patients: 60.4 years)^324^ and related to cardiovascular and all‐cause death (mean age of patients: 61.7 years).^325^ In the management of older patients with diabetes, TIR can also be used as a supplementary target for evaluating the glycemic control level, but such use remains to be supported by studies in older patients with diabetes. The definition of TIR goal should consider the individual differences and hypoglycemia risk of older patients with diabetes and needs support from further evidence‐based medical proofs. Research show a strong correlation between TIR and TAR in T1DM, but a weak correlation with TBR. For older patients using automated insulin delivery systems, prioritizing the reduction of TBR is recommended to decrease the risk of hypoglycemia.^326^ In addition to affecting glycemic control, CGM offers other benefits for older diabetes patients, such as continuous sharing of glucose results with family or caregivers, reducing the need for fingerstick glucose testing, especially for patients with physical disabilities, cognitive impairments, or visual impairments. For patients with reduced or impaired hypoglycemia awareness, CGM can help them detect low blood sugar episodes.^327^

### 18.2 Injection techniques

Correct subcutaneous insulin injection techniques include changing the needle after each injection, choosing an appropriate injection site, changing the injection site, taking care of the injection site appropriately, and avoiding intramuscular injection. The recommended injection sites include the abdomen, thigh, buttock, and upper arm. Long‐term injection at the same site may cause local lipohypertrophy or lipoatrophy^328^; thus the injection site should be changed regularly and alternately. A survey conducted by the China IT IMPROVE study on Chinese diabetes patients, doctors, and nurses revealed that 34.7% of patients experienced fat hypertrophy at injection sites. The reuse of needles and improper rotation of injection sites increased the risk of fat hypertrophy by 3.15 times and 1.27 times, respectively, and also increased the risk of uncontrolled HbA_1c_ levels.^329^

In older diabetes patients with conditions such as dementia, vision loss, neuropathy, poor mobility, and limited finger dexterity, there is an increased risk of missed or incorrectly administered insulin injections, and doctors are unable to accurately know the actual status of the patient's insulin injections. With continuous improvements in insulin injection pens, additional features have been developed and applied. Memory functions can record the dose and time of the last insulin injection, improving adherence to insulin therapy.^330^ By connecting to smart devices such as smartphones with close‐range wireless communication support, insulin injection records can be transmitted, visualized through applications, and shared. A prospective clinical study suggests that connected insulin pens can significantly reduce insulin injection omissions by 43%, improve TIR, and reduce TAR and TBR.^331^

Needle‐free injection technology is one of the recommended injection methods in the “Chinese Diabetes Drug Injection Technology Guidelines.” It can alleviate patients' needle phobia associated with traditional needle injection pens, reduce injection pain, thereby improving patient compliance and enhancing blood glucose control.^332^ In addition, needle‐free injection can reduce adverse reactions associated with needle injections, such as subcutaneous nodules, fat hypertrophy, or atrophy, and reduce insulin dosage. It can be used conditionally for older diabetes patients in good (Group 1) and intermediate (Group 2) health. However, needle‐free injection is more complicated than insulin pen injection and it is necessary to be instructed by professional personnel and master the operation method before self‐injection. Patients who cannot self‐administer injections should be educated to actively assist by family members or consider alternative injection methods.

### 18.3 Insulin pump

Insulin pump is an insulin delivery device controlled by artificial intelligence. It can carry out continuous subcutaneous insulin injection and maximally simulate the physiological secretion of insulin to facilitate blood glucose management. For older patients with diabetes, especially T1DM, if multi‐dose subcutaneous insulin injection therapy is associated with great glycemic variability and high hypoglycemia risk, insulin pump will be suggested. Patients who struggle with the precise adjustment of insulin dosage with multiple subcutaneous insulin injections may also consider the application of an insulin pump. Research suggests that in older T2DM patients, compared to multiple insulin injections, the use of an insulin pump effectively controls blood glucose with good safety and patient satisfaction.^333^ Studies confirm the safety and efficacy of the automatic insulin delivery system in T2DM patients with an average age exceeding 60 years.^334^

Cognitive decline, visual acuity decrease, finger dexterity decrease, lack of assistance from others, and lack of insulin pump knowledge may all be factors that limit the use of insulin pump in older patients with diabetes.^335^ Clinicians should determine whether to adopt insulin pump therapy based on patients' individual condition and adequately educate patients and their families on knowledge about insulin pump, including the setting of insulin pump, how to handle alarms, and potential complications, etc. A good assistance pattern should be established to handle the requirement rapidly and effectively for insulin pump adjustment, so as to avoid insulin pump–related acute adverse events.

## 19 CHAPTER 17: DIABETES IN THE OLDER PATIENTS AND TRADITIONAL CHINESE MEDICINE (TCM)

In Chinese medicine, diabetes belongs to the category of “Xiaoke disease (disease with symptoms of frequent drinking and urination)” and has a long history of traditional medical treatment in China. According to TCM, its basic pathogenesis is “Yin deficiency and dryness‐heat” and involving diseases in the lungs, stomach, kidneys, etc. Diabetes is classically divided into three types: upper, middle, and lower Xiaoke, and each has characteristic symptoms. The upper type is characterized by excessive thirst, the middle by excessive hunger, and the lower by excessive urination. TCM has a certain efficacy in the treatment of diabetes and its complications; however, because older patients often have declined organ function, multiple complications and comorbidities, and the combined use of Chinese and Western medicines is complex, patients should receive TCM treatment or combined traditional Chinese and Western medicine treatment under the guidance of a professional TCM practitioner and pay attention to medication safety during the treatment.

### 19.1 The guideline writing group

Qi Pan (Department of Endocrinology, Beijing Hospital, National Center of Gerontology).

Mingqun Deng (Department of Endocrinology, Beijing Hospital, National Center of Gerontology).

Ruiqi Yu (Department of Endocrinology, Peking Union Medical College Hospital, Chinese Academy of Medical Sciences).

### 19.2 Members of the expert group (Rank by surname alphabetic)

Hong Chen (Department of Endocrinology, Zhujiang Hospital of Southern Medical University).

Li Chen (Department of Endocrinology, Qilu Hospital of Shandong University).

Liming Chen (Department of Endocrinology, Tianjin Medical University Chu Hsien‐I Memorial Hospital).

Youxin Chen (Department of Ophthalmology, Peking Union Medical College Hospital, Chinese Academy of Medical Sciences).

Jingtao Dou (Department of Endocrinology, First Medical Center, Chinese PLA General Hospital).

Jianling Du (Department of Endocrinology, The First Affiliated Hospital of Dalian Medical University).

Binhong Duan (Department of Endocrinology, Heilongjiang Provincial Hospital).

Lixin Guo (Department of Endocrinology, Beijing Hospital, National Center of Gerontology).

Tianpei Hong (Department of Endocrinology, Peking University Third Hospital).

Xinguo Hou (Department of Endocrinology, Qilu Hospital of Shandong University).

Ji Hu (Department of Endocrinology, The Second Affiliated Hospital of Suzhou University).

Linong Ji (Department of Endocrinology, Peking University People's Hospital).

Qiuhe Ji (Endocrine and Metabolic Disease Hospital, Xi’an International Medical Center Hospital).

Sheng Jiang (Department of Endocrinology, The First Affiliated Hospital of Xinjiang Medical University).

Xin Jiang (Department of Geriatrics, Shenzhen People's Hospital).

Lin Kang (Department of Geriatrics, Peking Union Medical College Hospital, Chinese Academy of Medical Sciences).

Hongyu Kuang (Department of Endocrinology, The First Affiliated Hospital of Harbin Medical University).

Jian Kuang (Department of Endocrinology, Guangdong Provincial People's Hospital).

Minxiang Lei (Department of Endocrinology, Xiangya Hospital Central South University).

Chunlin Li (Department of Endocrinology, Second Medical Center, Chinese PLA General Hospital).

Quanmin Li (Department of Endocrinology, PLA Rocket Force Characteristic Medical Center).

Xing Li (Department of Endocrinology, Second Hospital of Shanxi Medical University).

Yanfeng Li (Department of Neurology, Peking Union Medical College Hospital, Chinese Academy of Medical Sciences).

Yiming Li (Department of Endocrinology, Huashan Hospital Fudan University).

Yong Li (Department of Cardiovascular Medicine, Huashan Hospital Fudan University).

Yuzhen Liang (Department of Endocrinology, Second Affiliated Hospital of Guangxi Medical University).

Xiahong Lin (Department of Endocrinology, The Seventh Affiliated Hospital, Sun Yat‐sen University).

Jing Liu (Department of Endocrinology, Gansu Provincial People's Hospital).

Youshuo Liu (Department of Geriatrics, The Second Xiangya Hospital of Central South University).

Bin Lu (Department of Endocrinology, Huadong Hospital Affiliated to Fudan University).

Yingli Lu (Department of Endocrinology, Shanghai Ninth People's Hospital, Shanghai JiaoTong University School of Medicine).

Wenshan Lv (Department of Endocrinology, The Affiliated Hospital of Qingdao University).

Yiming Mu (Department of Endocrinology, First Medical Center, Chinese PLA General Hospital).

Qi Pan (Department of Endocrinology, Beijing Hospital, National Center of Gerontology).

Guijun Qin (Department of Endocrinology, The First Affiliated Hospital of Zhengzhou University).

Yunfeng Shen (Department of Endocrinology, The Second Affiliated Hospital of Nanchang University).

Hong Shi (Department of Geriatrics, Beijing Hospital, National Center for Geriatrics).

Guangyao Song (Department of Endocrinology, Hebei General Hospital).

Nanwei Tong (Department of Endocrinology, West China Hospital of Sichuan University).

Jianye Wang (Department of Urology, Beijing Hospital, National Center for Geriatrics, Institute of Geriatrics, Chinese Academy of Medical Sciences).

Shidong Wang (Department of Endocrinology, Dongzhimen Hospital, Beijing University of Chinese Medicine).

Yangang Wang (Department of Endocrinology, The Affiliated Hospital of Qingdao University).

Zhengzhen Wang (School of Sports Medicine and Rehabilitation, Beijing Sport University).

Jing Wu (Department of Endocrinology, Xiangya Hospital Central South University).

Tianfeng Wu (Department of Endocrinology, Zhejiang Hospital).

Xiaohong Wu (Department of Endocrinology, Zhejiang Provincial People's Hospital).

Jianzhong Xiao (Department of Endocrinology, Beijing Tsinghua ChangGung Hospital).

Xinhua Xiao (Department of Endocrinology, Peking Union Medical College Hospital, Chinese Academy of Medical Sciences).

Xiaomin Xie (Department of Endocrinology, The First People's Hospital of Yinchuan).

Jing Xu (Department of Endocrinology, The Second Affiliated Hospital of Xi'an Jiaotong University).

Xiangjin Xu (Department of Endocrinology, Liberation Army Hospital 900).

YanCheng Xu (Department of Endocrinology, Zhongnan Hospital of Wuhan University).

Yong Xu (Department of Endocrinology, The Affiliated Hospital of Southwest Medical University).

Yushan Xu (Department of Endocrinology, First Affiliated Hospital of Kunming Medical University).

Zhangrong Xu (Department of Endocrinology, PLA Strategic Support Forces Characteristic Medical Center).

Yaoming Xue (Department of Endocrinology, Nanfang Hospital).

Chuan Yang (Department of Endocrinology, Sun Yat‐sen Memorial Hospital, Sun Yat‐sen University).

Jing Yang (Department of Endocrinology, First Hospital of Shanxi Medical University).

Tao Yang (Department of Endocrinology, The First Affiliated Hospital With Nanjing Medical University).

Ying Yang (Department of Endocrinology, Yunnan University Affiliated Hospital).

Xuefeng Yu (Department of Endocrinology, Tongji Hospital, Tongji Medical College of Huazhong University of Science and Technology).

Huijuan Yuan (Department of Endocrinology, Henan Provincial People's Hospital).

Bo Zhang (Department of Endocrinology, China‐Japan Friendship Hospital).

Cuntai Zhang (Department of Geriatrics, Tongji Hospital, Tongji Medical College of Huazhong University of Science and Technology).

Junqing Zhang (Department of Endocrinology, Peking University First Hospital).

Qiu Zhang (Department of Endocrinology, The First Affiliated Hospital of Anhui Medical University).

Jiajun Zhao (Department of Endocrinology, Shandong Provincial Hospital).

## AUTHOR CONTRIBUTIONS

Initiation and organization this guideline: Lixin Guo, Xinhua Xiao, National Center of Gerontology, Chinese Society of Geriatrics, Diabetes Professional Committee of Chinese Aging Well Association. Writing the initial draft: Qi Pan, Mingqun Deng, Ruiqi Yu. Preparation and presentation of the published work: Qi Pan, Mingqun Deng, Ruiqi Yu. Critical review and revision: Lixin Guo, Xinhua Xiao, and The Expert Group.

## FUNDING INFORMATION

None.

## CONFLICT OF INTEREST STATEMENT

All authors have nothing to disclose.

## APPENDIX A

### A.1

ADL scale.

**Table:**

ADL	Independent	Dependent
Feeding		
Bathing		
Dressing		
Going to Toilet		
Transfer		
Continence		

*Note*: ADL is activities of daily living. Number of impaired ADLs:________(Determined by assessment of the six aforementioned activities. If an individual is unable to perform an activity independently and requires assistance from others, it is to be considered as one impairment. The total number of impairments is calculated accordingly).

## APPENDIX B

### B.1

IADL Scale.

**Table:**

IADL	Independent	Dependent
Mode of transportation	Travels independently or arranges own travel via taxi or travels on public transportation when assisted or accompanied by another	Travel limited to taxi or automobile with assistance of another or does not travel at all
Food preparation	Plans, prepares, and serves adequate meals independently	Prepares adequate meals if supplied with ingredients or needs to have meals prepared and served
Responsibility for own medications	Is responsible for taking medication in correct dosages at correct time	Takes responsibility if medication is prepared in advance in separate dosages, or not capable of dispensing own medication
Laundry	Does personal laundry completely or launders small items	All laundry must be done by others
Ability to use telephone	Operates telephone on own initiative, dials a few well‐known numbers, or answers telephone but does not dial	Does not use telephone at all
Ability to handle finances	Manages financial matters independently, or manages day‐to‐day purchases, but needs help with banking, major purchases, etc	Incapable of handling money
Shopping	Takes care of all shopping needs independently	Shops independently for small purchases or needs to be accompanied on any shopping trip, or completely unable to shop
Housekeeping	Maintains house alone, performs light daily tasks, or performs light daily tasks but needs help with all home maintenance tasks	Does not participate in any housekeeping tasks

*Note*: IADL is instrumental activities of daily living. Number of impaired IADLs:________(Determined by assessment the aforementioned eight activities. If an individual is unable to perform an activity independently and requires assistance from others, it is to be considered as one impairment. The total number of impairments is calculated accordingly).
